# Ergot Alkaloids (Re)generate New Leads as Antiparasitics

**DOI:** 10.1371/journal.pntd.0004063

**Published:** 2015-09-14

**Authors:** John D. Chan, Prince N. Agbedanu, Thomas Grab, Mostafa Zamanian, Peter I. Dosa, Timothy A. Day, Jonathan S. Marchant

**Affiliations:** 1 Department of Pharmacology, University of Minnesota, Minneapolis, Minnesota, United States of America; 2 Department of Biomedical Sciences, Iowa State University, Ames, Iowa, United States of America; 3 Institute for Therapeutics Discovery and Development, University of Minnesota, Minneapolis, Minnesota, United States of America; 4 Stem Cell Institute, University of Minnesota, Minneapolis, Minnesota, United States of America; McGill University, CANADA

## Abstract

Praziquantel (PZQ) is a key therapy for treatment of parasitic flatworm infections of humans and livestock, but the mechanism of action of this drug is unresolved. Resolving PZQ-engaged targets and effectors is important for identifying new druggable pathways that may yield novel antiparasitic agents. Here we use functional, genetic and pharmacological approaches to reveal that serotonergic signals antagonize PZQ action *in vivo*. Exogenous 5-hydroxytryptamine (5-HT) rescued PZQ-evoked polarity and mobility defects in free-living planarian flatworms. In contrast, knockdown of a prevalently expressed planarian 5-HT receptor potentiated or phenocopied PZQ action in different functional assays. Subsequent screening of serotonergic ligands revealed that several ergot alkaloids possessed broad efficacy at modulating regenerative outcomes and the mobility of both free living and parasitic flatworms. Ergot alkaloids that phenocopied PZQ in regenerative assays to cause bipolar regeneration exhibited structural modifications consistent with serotonergic blockade. These data suggest that serotonergic activation blocks PZQ action *in vivo*, while serotonergic antagonists phenocopy PZQ action. Importantly these studies identify the ergot alkaloid scaffold as a promising structural framework for designing potent agents targeting parasitic bioaminergic G protein coupled receptors.

## Author Summary

The parasitic infection schistosomiasis afflicts millions of people worldwide and is clinically treated using a single drug, praziquantel (PZQ). Despite the fact that PZQ has served as a stalwart anthelmintic for decades, the molecular basis of action of this clinical agent is poorly understood. This lack of mechanistic information impedes the rational design of alternative therapies and highlights the need for new approaches for studying the target(s) and effectors engaged by PZQ *in vivo*. Here, we exploit the predictive phenology between free-living planarian regenerative screens and parasitic neuromuscular physiology to reveal a broad efficacy of ergot alkaloids in phenocopying the action of PZQ. In planarian regenerative screens, data highlight structural features of the ergoline scaffold that yield specific regenerative effects to promote or inhibit head regeneration. Ergot alkaloids with efficacy in regenerative assays were also found to modulate the contractility of schistosomules. Overall, these data highlight a possible therapeutic potential of ergot alkaloids as antischistosomals and the action of PZQ as an ergomimetic.

## Introduction

Schistosomiasis is a neglected tropical disease that infects over 200 million people worldwide, burdening economies with an annual loss of several million disability-adjusted life years [1–3]. The disease is caused by parasitic flatworms of the genus *Schistosoma* and treatment is largely reliant on a single drug—praziquantel (PZQ), used clinically for over 30 years [4–6]. PZQ is a synthetic tetracyclic tetrahydroisoquinoline that was initially developed by Merck while screening for compounds with tranquilizer properties, and arose from a compound that lacked sedative properties but was remarkably effective against parasitic flatworms [7,8]. PZQ has shown remarkable durability compared with other anthelmintics, but incidences of decreased PZQ efficacy have been reported in both the laboratory [9–11] and the field [12,13], raising concerns that PZQ-resistant strains of schistosomiasis may emerge especially as eradication initiatives increase distribution of this drug [4]. Development of alternative therapies to PZQ has been hampered by the fact that the mechanism of action of PZQ remains unresolved and rationally designed derivatives of PZQ typically prove less efficacious [7,14,15]. These longstanding roadblocks impair the iteration of next generation antischistosomals needed to counter the likely emergence of PZQ-resistant isolates [16]. Resolution of the pathways engaged by PZQ *in vivo* is therefore a key priority.

**Fig 1:**
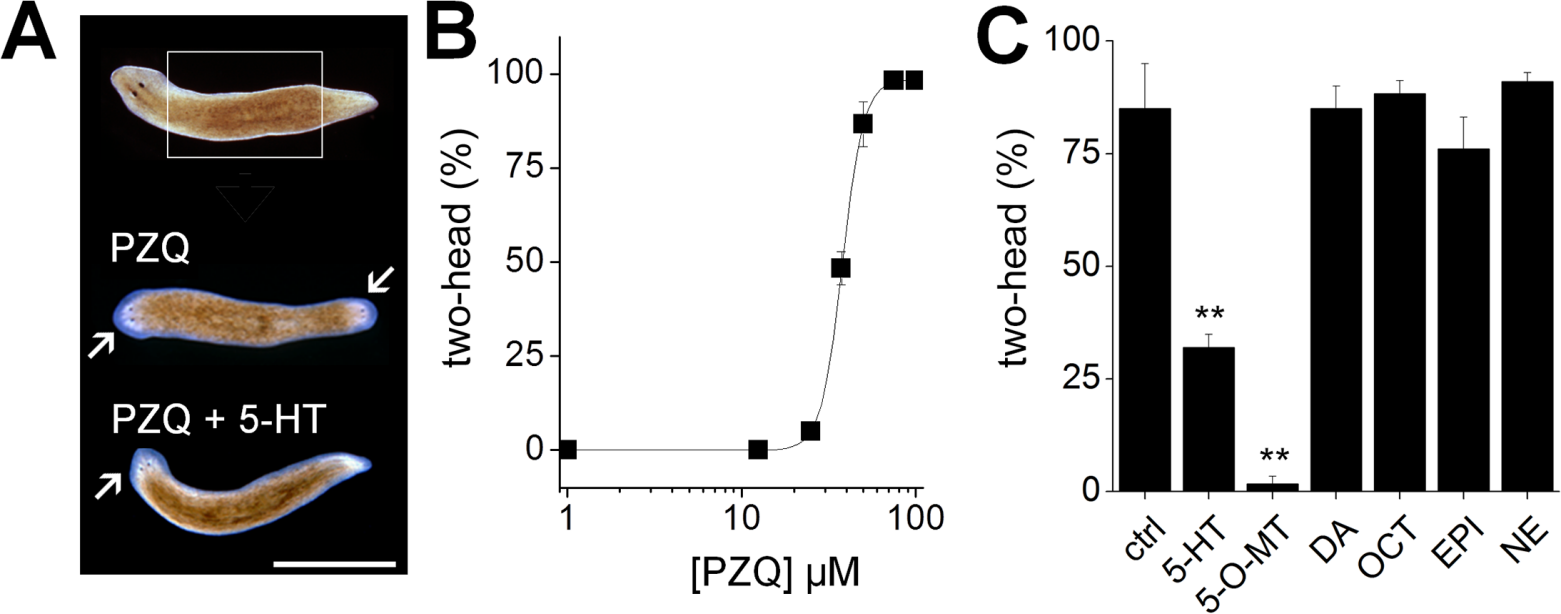
5-HT reverses PZQ action on regenerative polarity. (**A**) Regenerative phenotypes (scored after 7 days) from excised trunk fragments (top, boxed region) exposed to either PZQ alone (75μM, 24hrs), or PZQ (75μM) and 5-HT (250μM, 24hrs). Original anterior of the worm oriented to the left, head structures are arrowed. Scalebar, 4mm. (**B**) Dose response relationship for the effect of PZQ on regenerative bipolarity. (**C**) Reversal of PZQ-evoked bipolarity (75μM, 24hrs) by simultaneous incubation with 5-HT but not other neurotransmitters (dopamine (DA), octopamine (OCT), epinephrine (EPI), norepinephrine (NE), at a final concentration of 250μM). The analogue O-methylserotonin (*O*-MT) was used at a final concentration of 100μM. **, p <0.01.

A fresh perspective toward this problem comes from the discovery of an unusual axis-duplicating effect of PZQ during regeneration of free-living planarian flatworms [17,18]. The striking phenotype of PZQ-evoked bipolarity (Fig 1A), coupled with the genetic tractability of this system for RNAi [19] and a retained predictive value against parasitic flatworms [20] establishes a novel platform for identifying relevant *in vivo* effectors of PZQ action based on the molecular phenology between these systems. In planarian regenerative screens, the bipolarizing efficacy of PZQ depends on a coupling of voltage-operated Ca^2+^ channels to bioaminergic signals [20], which likely regulate polarity signaling from flatworm muscle cells to coordinate regenerative outcomes [21].

Here, we apply genetic and pharmacological approaches to dissect our observation that activation of serotonergic signaling in the planarian *Dugesia japonica* blocks the bipolarizing ability of PZQ. This effect is shown to be unique to serotonin (5-HT), and highlights the importance of characterizing serotonergic receptors to identify 5-HT blockers that could potentiate, or phenocopy, PZQ action. Intriguingly, serotonergic screens highlight ergot alkaloids as a class of compounds that potently and penetrantly miscued planarian regeneration and schistosomule muscle function, with structure activity insight from active compounds highlighting modifications of the ergot scaffold predictive for flatworm efficacy. Based on these data, we contend that the ergot alkaloid scaffold merits further exploration to yield novel chemotherapeutics with selective efficacy against parasite musculature.

## Results

### The bipolarizing action of PZQ is reversed by 5-HT

**Fig 2:**
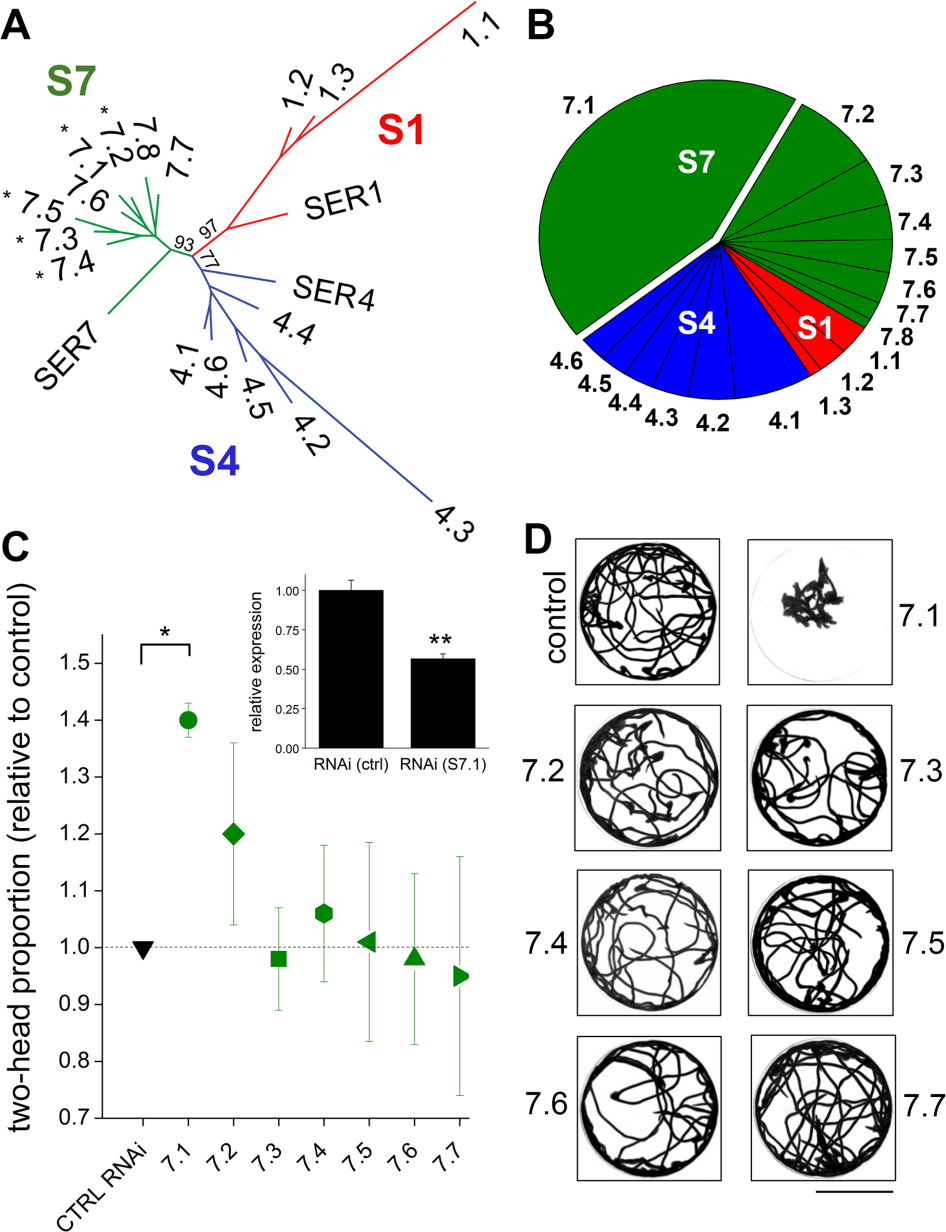
Analysis of serotonergic receptors in *Dugesia japonica*. (**A**) Unrooted maximum likelihood tree (PhyML 500 bootstrap replicates) of predicted *D*. *japonica* serotonin GpCR protein sequences (Dataset A and B in S1 Text) shows the distribution of 17 planarian receptors in S1-like (red), S4-like (blue) and S7-like clades (green). *C*. *elegans* sequences used for comparison are CeSER1a (O17470, SER1), CeSER4 (G5EGH0, SER4) and CeSER7a (Q22895, SER7). Previously cloned planarian 5-HTR sequences (*) are renamed as follows: S7.1 = 5HTLpla4 [23], DtSER1 [25]; S7.2 = 5HTLpla1 [23]; S7.3 = 5HTLpla2 [23]; S7.4 = DjSer7 [22]; S7.5 = 5HTLpla3 [23]. (**B**) Pie chart showing relative abundance of 5-HT GpCR transcripts as reflected by FPKM values. S7.1 represented the most abundant 5-HT receptor (~44% of all transcripts) and S7 the most abundant clade (~70% of all transcripts). (**C**) Effect of RNAi against individual S7 receptors on drug-evoked bipolarity. Data are expressed as the proportion of two-headed worms evoked by submaximal PZQ in RNAi worms (75μM) relative to a control RNAi cohort (*Smed*-six-1 RNAi). Data are not presented for S7.8 (the least abundant S7 receptor) as attempts to amplify this sequence were unsuccessful. Data represent mean± standard error of 3 to 7 independent knock-down cycles (*, p<0.02). *Inset*, qPCR analysis of S7.1 expression levels following RNAi relative to control RNAi cohorts (**, p<0.002). (**D**) Effect of RNAi targeting individual S7 receptors on intact worm mobility. Minimal intensity projection image represents motion of 10 worms over a 2 minute period within an illuminated watchglass. Scalebar, 25mm.

**Fig 3:**
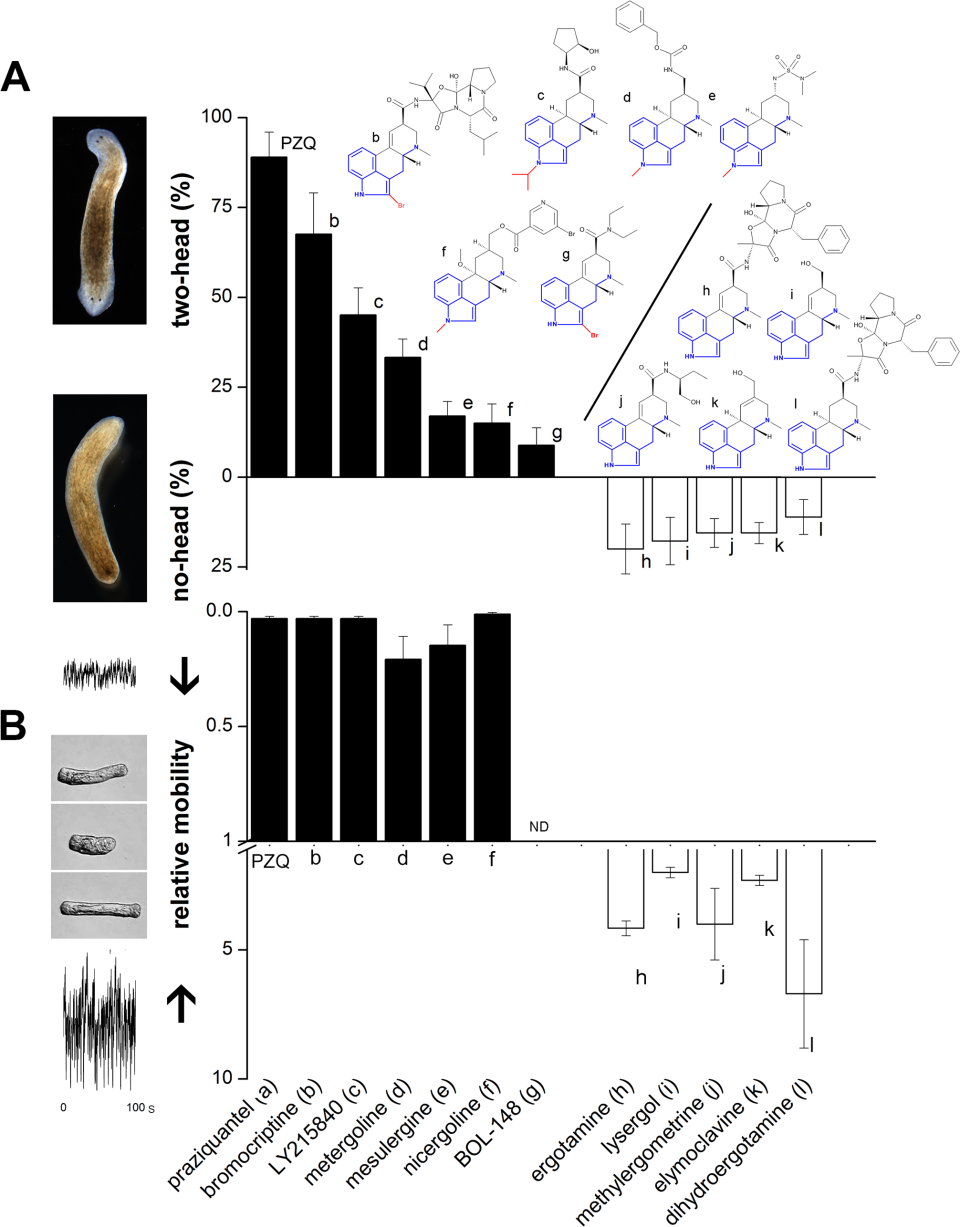
Effect of various ergot alkaloids on planarian regeneration and schistosomule contractility. (**A**) Effect of different ergot alkaloids on planarian regenerative polarity. Compounds caused either bipolar regeneration (‘two-head’, solid) or no-headed regenerants (open). Illustrative phenotypes are indicated (left) resulting from exposure to LY215840 (top) or ergotamine (bottom). Structures of individual compounds (‘a’ through ‘l’) are indicated. Indole ring modifications are highlighted in red, and shared structure with 5-HT shown in blue. Concentrations used for regenerative assay (24/48hrs) were: ‘a’ (75μM), ‘b’ (1.5μM), ‘c’ (25μM), ‘d’ (1μM), ‘e’(10μM), ‘f’ (2μM), ‘g’ (1μM), ‘h’ (1μM), ‘I’ (5μM), ‘j’ (10μM), ‘k’ (10μM), ‘l’ (10μM). (**B**) Effect of the same ergot alkaloids on contractile activity of schistosomules, with compounds grouped as in (A). Decreased (solid) and increased (open) mobility are expressed relative to controls (set as ‘1’). Body length versus time plots (left) were resolved for individual schistosomules treated with small molecules. Example traces are shown for LY215840 (top) and ergotamine (bottom), relative to control. Drug concentrations (30 minute exposures) were: ‘a’ (10μM), ‘b’ (10μM), ‘c’ (50μM), ‘d’ (50μM), ‘e’(25μM), ‘f’ (25μM), ‘g’ (ND = not done), ‘h’ (0.5μM), ‘I’ (10μM), ‘j’ (5μM), ‘k’ (1μM), ‘l’ (0.5μM). Data for PZQ and bromocriptine are from [20].

Exposure of regenerating planarian (*D*. *japonica*) trunk fragments to praziquantel (PZQ) yielded two-headed worms (Fig 1A, [17]), an effect never observed in the absence of drug exposure. This effect was dose-dependent (EC_50_ 38±3.6μM, Fig 1B), with maximal doses being completely penetrant [17]. Strikingly, the bipolarizing action of PZQ was blocked by co-incubation with 5-HT, or the analogue *O*-methylserotonin (*O*-MT), but not by co-incubation with other bioaminergic neurotransmitters (Fig 1C). As this result suggests serotonergic signals functionally oppose PZQ action, we implemented genetic (Fig 2) and pharmacological strategies (Fig 3) to interrogate 5-HT signaling pathways in planarians.

### RNAi of 5-HT receptors potentiates PZQ action

To enable interrogation of 5-HT receptor function by *in vivo* RNAi, we generated a *de novo* transcriptome assembly for *D*. *japonica* (see Methods) to allow a comprehensive bioinformatic identification of 5-HT receptors in this system (Fig 2A). A total of 17 predicted serotonergic G protein coupled receptor (GpCR) sequences were identified based upon homology to previously identified sequences [22–24]. These putative 5-HT receptors clustered into three discrete clades (S1-, S4- and S7-like, Fig 2A) defined by homology with *C*. *elegans* serotonin receptors (SER1, SER4 & SER7) [24]. Previously identified planarian 5-HT receptors (5HTLpla1-4, DjSER-7, DtSer1 [22,23,25]) all localized within the Ser-7 clade.

To simplify nomenclature for these sequences, we assigned names to each receptor based on these three clades and transcript abundance within each grouping (from FPKM values, fragments per kilobase of transcript per million mapped reads), such that the most abundant transcript in the S7 clade was named S7.1 and the least abundant of the eight transcripts was designated as S7.8. Comparison of FPKM values for all these sequences revealed that S7.1 was the most abundantly expressed 5-HT receptor in this system (Fig 2B), accounting for ~40% of the total FPKM values assigned to all predicted serotonergic receptors. As the most abundant receptor, S7.1 had previously been cloned by degenerate PCR (5HTLpla4, DtSer1 [23,25]) and recently demonstrated to couple to cAMP generation [25]. Expression levels of S7.1 mRNA changed during regeneration [23], and we observed increased FPKM values for S7.1 at early regenerative timepoints (Fig A in S1 Text). Although prediction of the S1, S4 and S7-like sequences as serotonergic GpCRs is based on specific sequence features known to be important for 5-HT binding (see below), as well as overall homology to other serotonin receptors (Fig B in S1 Text), we do note that both the planarian receptor (S7.4, DjSER-7 [22]) and a schistosome S7-like receptor have been successfully deorphanized following heterologous expression and shown to respond to 5-HT [25,26].

Alignment of the planarian sequences with human bioaminergic GpCR sequences (Table 1) revealed conservation of key residues within the orthosteric binding pocket known to be important for ligand binding. With reference to molecular docking studies of 5-HT into crystal structures of human 5-HT_1B_ (and 5-HT_2B_) receptors [27], these include (i) a salt bridge between the amino group of 5-HT and D3.32 in the 5-HT_1B_ receptor (itself stabilized by Y7.43, Ballesteros & Weinstein numbering [28]), (ii) a hydrogen bond from T3.37 to the indole (N-H) hydrogen of 5-HT, and (iii) a hydrophobic cleft formed by contributions from (W6.48, F6.51, F6.52, C3.36 and I3.33). All these residues are well conserved in the planarian receptor sequences (Table 1). Notably, bioaminergic receptors that respond to different ligands (e.g. dopamine, adrenaline, histamine) present a more polar interface at resides 5.42 and 5.46, whereas human 5-HT sequences present no more than one polar residue ([27], Table 1). This feature has been suggested to facilitate interaction with the less polar indole group of 5-HT compared to the other bioaminergic transmitters [27]. The planarian sequences also conform to this principle with the combination of residues at this position being diagnostic of the three different clades of 5-HT receptor sequences (e.g. ‘AA’ for S7, ‘S/A, A/S for S4, ‘xT’ for S1, Table 1). Another notable feature of the planarian 5-HT groupings is receptor architecture, for example the spacing between these critical residues in helix 3 and helix 5, and helix 5 and helix 6 (Table 1) appears diagnostic of the different serotonergic clades. For example, the S7 clade exhibits a consistent spacing (~74 residues) between 3.37 and 5.42, and a shorter third intracellular loop between TM5 and TM6 compared with the other clades.

**Table 1:** Comparison of key residues within the orthosteric binding pocket of planarian 5-HT receptors. Table shows amino acid identity at indicated residues (column header, B&W nomenclature [28]) in individual S1, S4 and S7 planarian receptors. S1.1 and S4.2 were omitted as transcriptomic sequence represented only partial clones that did not contain the relevant sequence information. Corresponding residues are shown for representative human GpCRs (bottom), including 5-HT receptors. Polarity of residue at 5.42 and 5.46 are highlighted (italics, non-polar; underlined, polar). Residues at 5.42 and 5.46 are thought to be less polar in 5-HT receptors compared with receptors with different bioaminergic specificity. Numeric values depict amino acid spacing between indicated B&W residues: for example, 3.37 and 5.42 are separated by ~74 residues in S7 receptors. Accession numbers used for comparison: 5HTR1B (P28222), 5HTR2B (P41595), 5HTR6 (P50406), DRD3 (dopamine D3 receptor, P35462), ADRB2 (beta-2 adrenoceptor, P07550), HRH1 (histamine H1 receptor, P35367).

	3.32	3.33	3.36	3.37		5.42	5.46		6.48	6.51	6.52	7.43
**S1.2**	**D**	**V**	**T**	**S**	[96]	**A**	***T***	[204]	**Y**	**F**	**F**	**Y**
**S1.3**	**D**	**V**	**T**	**A**	[80]	**A**	***T***	[193]	**Y**	**F**	**F**	**Y**
**S4.1**	**D**	**V**	**C**	**S**	[81]	***S***	**A**	[350]	**W**	**F**	**F**	**Y**
**S4.3**	**D**	**V**	**C**	**T**	[81]	**A**	***S***	[306]	**W**	**F**	**G**	**Y**
**S4.4**	**D**	**V**	**C**	**S**	[81]	***S***	**A**	[166]	**W**	**F**	**F**	**Y**
**S4.5**	**D**	**V**	**C**	**T**	[80]	***S***	**A**	[247]	**W**	**F**	**G**	**Y**
**S4.6**	**D**	**V**	**C**	**S**	[81]	***S***	**A**	[230]	**W**	**F**	**F**	**Y**
**S7.1**	**D**	**V**	**C**	**T**	[74]	**A**	**A**	[116]	**W**	**F**	**F**	**Y**
**S7.2**	**D**	**V**	**C**	**T**	[74]	**A**	**A**	[117]	**W**	**F**	**F**	**Y**
**S7.3**	**D**	**V**	**C**	**T**	[74]	**A**	**A**	[115]	**W**	**F**	**F**	**Y**
**S7.4**	**D**	**V**	**C**	**T**	[74]	**A**	**A**	[175]	**W**	**F**	**F**	**Y**
**S7.5**	**D**	**V**	**C**	**T**	[74]	**A**	**A**	[187]	**W**	**F**	**F**	**Y**
**S7.6**	**D**	**V**	**C**	**S**	[74]	**A**	**A**	[116]	**W**	**F**	**F**	**Y**
**S7.7**	**D**	**V**	**C**	**T**	[74]	**A**	**A**	[124]	**W**	**F**	**F**	**Y**
**S7.8**	**D**	**I**	**C**	**T**	[73]	**A**	**A**	[151]	**W**	**F**	**F**	**Y**
**5HTR1B**	**D**	**I**	**C**	**T**	[77]	***S***	**A**	[108]	**W**	**F**	**F**	**Y**
**5HTR2B**	**D**	**V**	**S**	**T**	[80]	**G**	**A**	[111]	**W**	**F**	**F**	**Y**
**5HTR6**	**D**	**V**	**C**	**S**	[80]	**A**	***T***	[84]	**W**	**F**	**F**	**Y**
**DRD3**	**D**	**V**	**C**	**T**	[83]	***S***	***S***	[145]	**W**	**F**	**F**	**Y**
**ADRB2**	**D**	**V**	**V**	**T**	[84]	***S***	***S***	[78]	**Y**	**F**	**F**	**Y**
**HRH1**	**D**	**Y**	**S**	**T**	[[81]	***T***	***N***	[229]	**W**	**Y**	**F**	**Y**

As S7 represented the most abundantly expressed clade of 5-HT GpCRs, we proceeded to perform RNAi against each individual receptor. First, we screened for effects on PZQ-evoked bipolarity. RNAi-mediated suppression of *S7*.*1* potentiated the number of two-headed regenerants (82±6%) compared to the number of bipolar worms observed in control RNAi cohorts (59±5% at submaximal PZQ). Estimation of the effectiveness of knockdown of *S7*.*1* transcripts was assessed by qPCR analysis in the same cohorts used for the regenerative assays. These assays revealed a decrease of 43±3% of *S7*.*1* mRNA relative to controls (Fig 2C, inset).

Aside from the polarity effect on regenerating fragments, *S7*.*1* RNAi also impaired the movement of intact worms. Planarians subject to *S7*.*1* RNAi showed decreased mobility (Fig 2D), quantified by monitoring the distance traversed by *S7*.*1* RNAi worms (61±4mm, average of 10 worms, n = 3 independent RNAi cohorts) compared with controls (104±7mm) over the same time period (2 mins). RNAi targeting other receptors in the S7 clade failed to yield a clear defect. We conclude knockdown of S7.1 modulated both regenerative polarity and motility outcomes.

### Serotonergic ligands modulate planarian regenerative polarity

Next, we employed a pharmacological approach to manipulate serotonergic signals by screening agents with known affinity for serotonergic receptors. While diverse classes of serotonergic blockers caused regenerative bipolarity, the penetrance was typically much lower than seen with PZQ (Table A in S1 Text). Results with ergot alkaloids were however of interest. Ergot alkaloids are a historically important class of compounds that realize their effects because of the close structural similarity of the ergoline scaffold to bioaminergic transmitters. Numerous ergot compounds yielded regenerative phenotypes, either phenocopying PZQ to promote bipolar (‘two-head’) regeneration or inhibiting head regeneration (‘no-head’, Fig 3A), all at doses lower than PZQ.

This broad efficacy of ergot alkaloids as a chemical class permitted structural-activity insight into features associated with specific polarity effects: for example, all ergots that caused bipolarity were either alkylated on the indole nitrogen or halogenated at the adjacent 2-position (Fig 3A). In contrast, all ergots that inhibited head regeneration lacked such modifications (Fig 3A). Structural studies have shown that the indole N1 hydrogen forms a key hydrogen bond with a conserved threonine residue T3.37 [27] within the orthosteric binding pocket of 5-HT GpCRs that is likely important for receptor activation [27,29]. This residue is also well conserved in the planarian 5-HT receptors sequences (Table 1). Disruption of this interaction by receptor mutagenesis interferes with 5-HT receptor activation by ergot alkaloids [29]. Similarly, alkylation of ergot derivatives at the N1 position also can cause decreased receptor activation yielding compounds that act as 5-HT receptor antagonists [30]. Therefore, this structural feature of the bipolarizing ergot compounds suggests they work through serotonergic blockade. This is consistent with observations that (i) structurally diverse 5-HT antagonists cause bipolarity (Table A in S1 Text), (ii) the ergots that inhibited head regeneration act as 5-HT agonists in other systems [31–33], (iii) other drugs that stimulate 5-HT signaling (8-OH DPAT and fluoxetine) also block head regeneration and PZQ action [20], and (iv) RNAi of tryptophan hydroxylase (TPH) to decrease 5-HT levels potentiates PZQ action [20]. Therefore, these data show that PZQ action mimics the bipolarizing ability of serotonergic blockers, and is opposed by 5-HT agonists.

### Modulation of schistosomule contractility

The importance of identifying new drugs from planarian regenerative screens extends beyond basic science as planarian regenerative assays can predict the efficacy of compounds against parasitic worms [20]. Exploiting this phenology may assist discovery of new drug leads and targets for treating parasitic disease. Therefore, we were interested to assess whether the same set of compounds active in regeneration assays displayed activity against schistosomules, the immature form of parasitic schistosome flatworms that exist after penetration of host skin. Schistosomes display an endogenous contractile cycle permitting drug-evoked effects to be easily screened (paralysis versus stimulation of contractility, Fig 3B). In schistosome contractility assays, the compounds that caused planarian bipolarity all inhibited schistosomule motility (just like PZQ), whereas the compounds that inhibited planarian head regeneration caused the opposite effect, stimulating contractile activity (Fig 3B). Therefore, ergot alkaloids possess efficacy against schistosomules, with an action predictable by planarian polarity outcomes.

### Drugs that miscue polarity target the excitable cell niche

What is the molecular basis of this phenology between planarian regenerative polarity and schistosome motility? An appealing explanation relates to the recent identification of muscle cells as the coordinating nexus of positional signaling during planarian regeneration [21]. Specifically, a subepidermal population of myocytes was identified to coexpress all the relevant ‘position control genes’ known to regulate the planarian body plan, from which positionally appropriate transcriptional responses are engaged on injury [21]. This discovery is enlightening as it harbors the potential to rationalize a long literature on the effects of exogenous agents on regeneration dating back decades by suggesting that drugs which miscue regenerative patterning all possess a shared ability to modulate excitable cell physiology and perturb muscle function.

**Fig 4:**
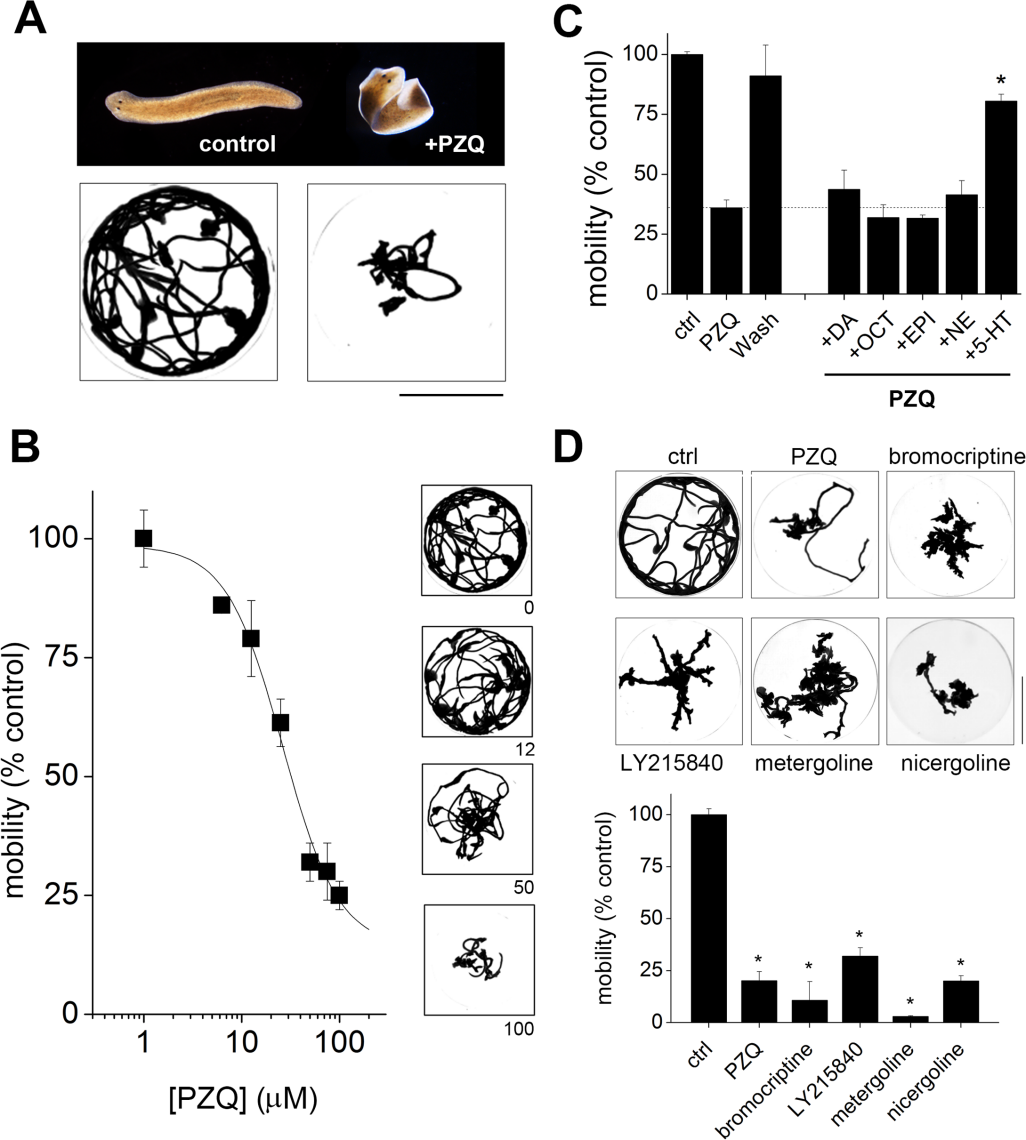
Bipolarizing compounds inhibit planarian mobility. (**A**) Top, reversible effect of PZQ on worm morphology (right) compared with control (left). Bottom, effect of PZQ on mobility of intact worms (75μM, 10mins incubation). Images show minimal intensity projections for 10 worms over 2 minutes. Scalebar, 25mm. (**B**) Dose response relationship for effect of PZQ on planarian mobility. Inset, minimal intensity projections at indicated doses (μM). (**C**) Antagonism of acute PZQ-evoked mobility defects in intact worms (75μM) by co-incubation with *O*-MT but not other neurotransmitters (all at 100μM). *, p < 0.01 relative to PZQ (dashed line). PZQ-evoked mobility defects are reversible on solution exchange (‘wash’). (**D**) Top, effects of bipolarizing ergot compounds on planarian mobility after 10min exposure. Bottom, quantification of the mobility effects evoked by different ergot alkaloids. Doses: PZQ (75μM), bromocriptine (2μM), LY215840 (1μM), metergoline (2μM), nicergoline (10μM). *, p < 0.01 relative to control.

Therefore, we examined whether PZQ and the ergot alkaloids discovered to miscue polarity, impacted planarian motility. Acute incubation of intact worms with PZQ caused worms to adopt a spastic, curled morphology with inhibitory effects on worm motion (Fig 4A). This effect was dose-dependent (Fig 4B), with a concentration-dependence similar to that observed for the (longer term) polarity effect (EC_50_ = 38±3.6μM for bipolarity in trunk fragments versus EC_50_ = 23±2.4μM for mobility in intact worms, Figs 1B and 4B) and reversible following drug removal (Fig 4C). Just as observed with PZQ-evoked bipolarity, the immobilizing action of PZQ was also reversed by co-incubation with *O*-MT, but not other bioaminergic neurotransmitters (Fig 4C). Finally, each of the ergot compounds discovered to cause bipolarity (Fig 3A) also inhibited planarian mobility (Fig 4D), underscoring the association between polarity-miscuing drugs and the ability to perturb flatworm muscle function.

### Effects of 5-HT on schistosomule contractility

**Fig 5:**
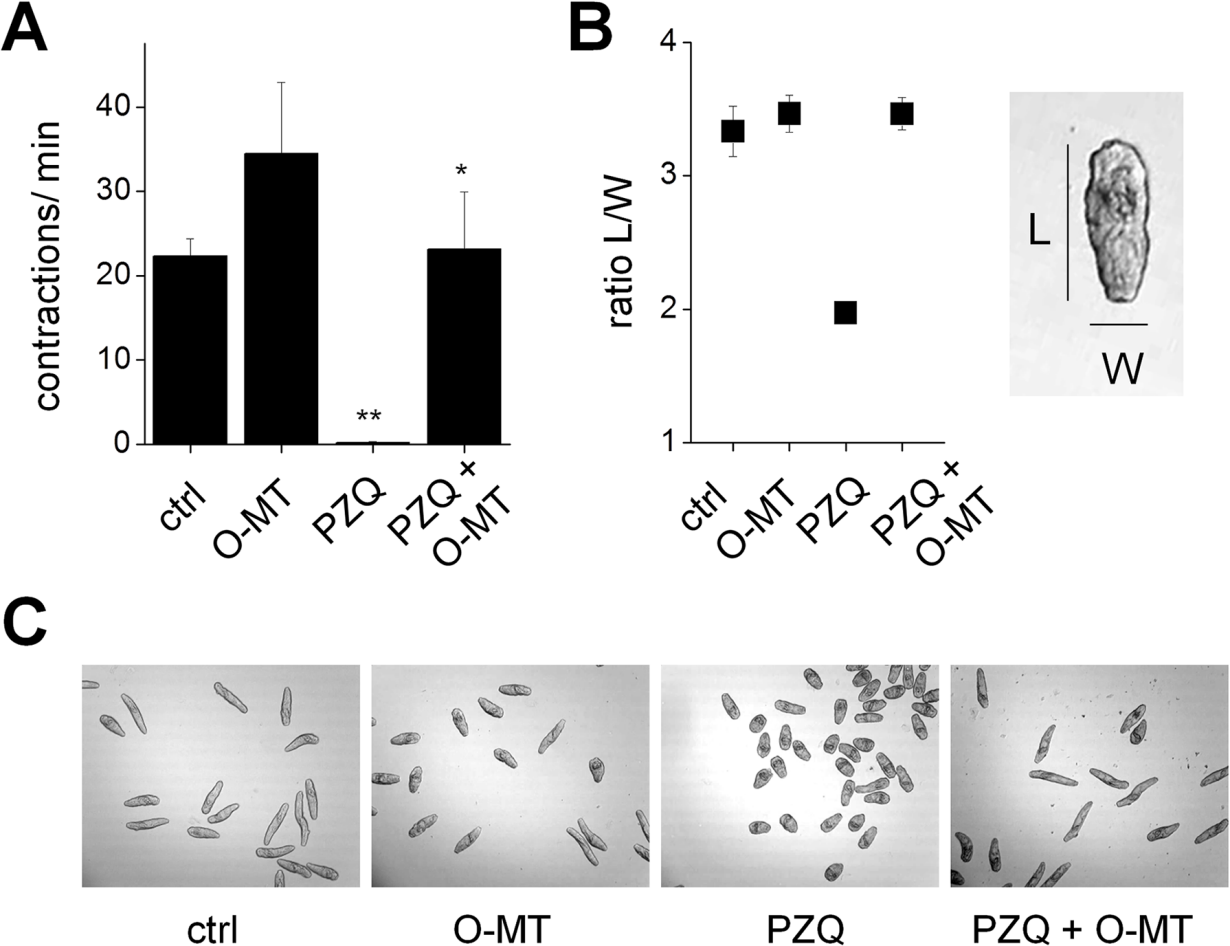
PZQ and 5-HT action against schistosomules. (**A**) Mobility of schistosomules (contractions per minute) exposed to the serotonergic agonist *o*-methylserotonin (*O*-MT, 1μM), praziquantel (PZQ, 50nM), or co-treated with PZQ (50nM) plus *O*-MT (1μM). **(B)** Measurement of schistosomule morphology changes in response to PZQ, as measured by the ratio of the worm’s average body length (‘L’) to width (‘W’) as depicted. **(C)** Phenotypes of schistosomules from experiments shown in B. *, p < 0.01 PZQ relative to *O-*MT + PZQ. **, p < 0.001 PZQ relative to control.

Finally, we returned to the fundamental observation of functional antagonism between serotonergic signals and PZQ action (Figs 1 and 4C). Does serotonergic activation modulate PZQ-evoked immobility in schistosomules? To address this, we examined the ability of exogenous O-MT to reverse PZQ-evoked effects on contractility (Fig 5A) and morphometry (Fig 5B and 5C). Addition of O-MT markedly ameliorated both the paralytic and compressed worm morphology resulting from PZQ exposure (Fig 5). Therefore, we conclude that PZQ action is functionally antagonized by serotonergic activation in schistosomule motility experiments, just as observed in planarian assays (Figs 1 and 4C).

## Discussion

### Serotonergic activation opposes PZQ action

**Fig 6:**
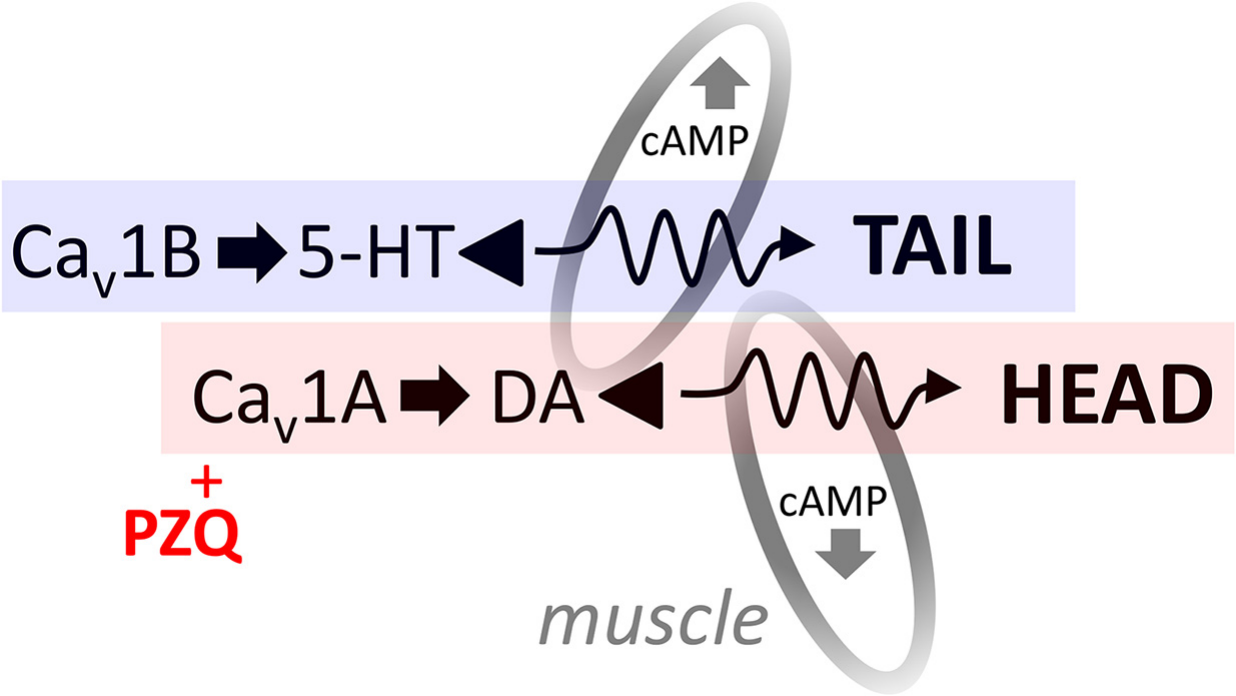
Proposed model of PZQ action. PZQ action in planarians has previously been shown to depend on Ca_v_1A and tyrosine hydroxylase (TH) functionality (red, [18,20]). Activity of this pathway is opposed by Ca_v_1B and tryptophan hydroxylase functionality (blue, [18,20]). We propose these pathways differentially regulate second messenger (cAMP) action, muscle function and—in planarians—‘head’ versus ‘tail’ specification. Here, we demonstrate the role of serotonergic antagonists as phenocopying PZQ action. This is most conservatively explained by functional antagonism of the opposing Cav1A/dopaminergic pathway, however the possibility that PZQ directly targets flatworm bioaminergic receptors as an ergomimetic cannot be excluded until heterologous expression of platyhelminth GpCRs is optimized.

The observations that serotonergic activation opposes, while serotonergic inhibition mimics PZQ action, reveal that serotonergic signaling is an important modulator of PZQ efficacy *in vivo*. We have previously suggested that PZQ engages dopaminergic pathways to subvert regeneration and it is noteworthy that both dopamine and serotonin regulate cAMP turnover with opposing effects on flatworm musculature [34–36]. Levels of cAMP change during regeneration [37] and cAMP is a known mediator of flatworm muscle contraction [38]. Therefore, this ‘functional antagonism’ model (Fig 6, [20]) envisages opposing Ca^2+^ entry pathways (Ca_v_1A versus Ca_v_1B) coupling to discrete bioaminergic neurotransmitters that differentially couple to cAMP within the excitable cell niche. Functional opposition of these bioaminergic systems are well evidenced in many systems [39].

### Ergot alkaloids provide new leads for schistosomiasis

Importantly, we demonstrate here that ergot alkaloids are efficacious modulators of planarian regeneration and motility (Figs 3 and 4). These two phenotypes are linked as surprisingly planarian polarity genes localize in a supepidermal population of muscle cells [21]. Indeed, ergot alkaloids have a well appreciated ability to modulate smooth muscle contraction based on their bioaminergic mimicry, a property that underpins several of their applications in the clinic. Beyond this ability to regulate muscle (including opposing effects on flatworm musculature [34–36]), dopamine and serotonin also are known regulators of Wnt signaling. D_2_Rs selectively associate with both β-catenin (to inhibit Wnt signals [40]) and Ca_v_ channels (to regulate their expression [41]). 5-HT is a well-established wounding signal [42], long range messenger involved in regenerative proliferation [43,44] and a reciprocally permissive cue for Wnt signaling [45,46]. Such associations provide precedence for coupling bioaminergic activity to the more established players of planarian regenerative signaling that localize with a myocyte population likely regulated by bioaminergic cues. Possibly all drugs that miscue regenerative polarity share such a commonality of action on the excitable cell niche.

Planarian regenerative screens hold predictive significance for discovering new drug leads and targets in parasitic flatworms [20]. Given the ease of performing drug screens in free living planarians compared to their parasitic cousins, this could be a fruitful source of novel therapeutic leads. 5-HT signaling in parasitic schistosomes is an appealing choice for therapeutic intervention given the dynamic expression of serotonergic gene products across the parasite life cycle [47–49] and a clearly evidenced role for bioaminergic signals in regulating muscle [34–36]. Parasite survival within the host requires worm muscle functionality: for example, muscle activity appears to be required for female pairing within the male gynecophoric canal, egg production and maintaining adult worm residency within the mesenteric vasculature. Paralytic agents such as PZQ have been proposed to act as antischistosomals by causing immobilized worms to shift from the mesenteric veins to the liver where they are eliminated [50]. Therefore, miscuing muscle function through bioaminergic cues is a promising route for drug intervention. Our data, revealing an ergomimetic quality to PZQ action, provide impetus for considering ergot alkaloids as potential drug leads for manipulating bioaminergic GpCRs to provide next generation antischistosomals [51]. Ergot alkaloids have been used clinically in a range of applications (migraine, obstetrics, Parkinson’s disease, diabetes), although owing to their broad GpCR binding profile they are often written off as problematic, ‘dirty’ compounds [30] and therefore often deliberately excluded from drug screens. However, this may be an oversight in the context of parasitic chemotherapy. Certain ergot compounds are penetrant and potent in planarian assays compared with PZQ. Further, the clear structural-activity principles emerging from our screen in free-living and parasitic worms (Fig 3) could illuminate structural differences in flatworm GpCR structure compared to their human hosts that may facilitate parasite targeting and mitigate host side effects. Based on our data from the planarian polarity and motility screens that are predictive of parasitic worm phenotypes, we contend that the ergot alkaloid scaffold merits further exploration by medicinal chemistry to identify novel chemotherapeutics with efficacy against parasite muscle.

## Materials and Methods

### Planarian husbandry

A clonal line of *Dugesia japonica* (GI strain) was maintained at room temperature and fed strained chicken liver puree once a week [52]. Regenerative assays were performed using 5 day-starved worms in pH-buffered Montjuïch salts (1.6mM NaCl, 1.0mM CaCl_2_, 1.0mM MgSO_4_, 0.1mM MgCl_2_, 0.1mM KCl,1.2mM NaHCO_3_, pH 7.4 buffered with 1.5mM HEPES) and regenerative phenotypes archived using a Zeiss Discovery v20 stereomicroscope and a QiCAM 12-bit cooled color CCD camera [52]. Data were analyzed using two-tailed, unpaired t-tests, and presented as mean ± standard error of the mean from at least three independent assays.

### Chemicals

Commercially available ergot alkaloids were sourced as follows: Sigma (bromocriptine, metergoline, nicergoline, ergotamine, dihydroergotamine); Tocris (LY215840, mesulergine, methylergometrine); THC Pharm (BOL-148, lysergol, elymoclavine). All other chemicals were from Sigma-Aldrich except where specified.

### Transcriptome assembly and bioinformatics

Total RNA from regenerating and intact *D*. *japonica* was harvested in Trizol and mRNA was purified by hybridization to oligo(dT) beads (Dynal). RNA-seq libraries were prepared according to the Illumina mRNA-Seq Sample Prep kit and Illumina TruSeq kit manufacturer protocols. Libraries were sequenced on Illumina HiSeq 2000 machines, producing 100bp paired end reads. Adapter sequences were trimmed and reads were passed through a sliding window quality filter (window size = 4, minimum average quality score = 25) using Trimmomatic version 0.22 [53]. Paired-end reads and singletons ≥ 50 bp in length were retained. Overlapping paired-end reads were merged using FLASH [54]. Surviving reads were combined and fed into the Trinity pipeline for *de novo* assembly [49]. Final assembly was carried out with a minimum k-mer coverage of 2 and the default k-mer size of 25. Complex graphs that proved unresolvable within a 6 hour window were manually excised to allow the assembly to proceed. The minimum contig or transcript length for both assembly pipelines was set to 100 nt. Candidate *D*. *japonica* 5-HT receptor sequences were selected based upon homology to receptors predicted in the planarian *Schmidtea mediterranea* [24]. Alignments were performed on predicted amino acid sequences in SeaView (version 4.5.1) using MUSCLE. Maximum likelihood phylogenies were generated using PhyML at 500 bootstrap replicates and visualized using FigTree (version 1.4.0).The depth of this resulting assembly proved comparable to transcriptomes generated for other planarian species [55,56], as well as the predicted open reading frames of the *S*. *mediterranea* genome [57], indicating that this resource is a reliable reference for the prediction and cloning of *D*. *japonica* gene products. The high level of coverage is evidenced by the fact that, of the 983 existing *D*. *japonica* nucleotide sequences manually cloned and deposited on NCBI, 982 are represented in our *de novo* assembly with a high degree of sequence identity. Reads were mapped onto the *de novo* assembly using RSEM [58] to obtain FPKM values reflecting transcript abundance. Sequences are provided as Supplementary material (Datasets A and B in S1 Text).

### Cloning strategies and RNAi analyses

Total RNA was isolated from 50 starved, intact planarians using TRIzol and poly-A purified using a NucleoTrap mRNA mini kit. cDNA was synthesized using the SuperScript III First-Strand Synthesis System (Invitrogen). Gene products were amplified by PCR (LA Taq polymerase), ligated into pGEM-T Easy (Promega) for sequencing, and subcloned into the IPTG-inducible pDONRdT7 RNAi vector transfected into RNase III deficient HT115 *E*. *coli*. *In vivo* RNAi was performed by feeding [52], and a *Schmidtea mediterranea* six-1 (*Smed*-six-1) construct, which did not yield a phenotype in *D*. *japonica*, was used as a negative control. Cohorts of worms were fed bacterially expressed dsRNA targeting individual 5-HT receptors or the negative control over a total of five feeding cycles (three RNAi feedings separated by 1–2 days, followed by amputation, regeneration, two more RNAi feedings, followed by excision of trunk fragments for regenerative assays). Targeted sequences for RNAi are provided in Supplementary Materials (Dataset A in S1 Text). Knockdown was assessed by quantitative RT-PCR. Total RNA was isolated from 10 intact worms, treated with DNAse I (Invitrogen) and cDNA synthesized using oligo(dT) primers and the SuperScript III First-Strand Synthesis System. Samples (10 intact worms) were homogenized in Trizol to extract total RNA which was treated with DNAse I (Life Technologies) and 500ng were used for cDNA synthesis using random hexamers (SuperScript III First-Strand Synthesis System, Life Technologies). No RT controls were produced by using the same procedure but substituting DEPC-treated water for SuperScript RT enzyme. TaqMan qPCR reactions were performed using custom-designed TaqMan Gene Expression Assays (Applied Biosystems). Assays were designed for GAPDH (F’ GCAAAAGACTGTTGATGGACCAT, R’ CACGGAAAGCCATTCCAGTTATTTT, probe sequence CCTCTGCCATCTCGCC) and 5-HTR 7.1 (F’ CAATCTATCAAGGTTAGCTATTCCATTCGA, R’ GCTCCCACAACGATAATAAAAAATATAATCCC, probe sequence ACCAACCGGATATTTT) and cycled in a StepOnePlus Real-Time PCR System (Applied Biosystems) at 50°C/2min, 95°C/10min, 40 cycles of 95°C/15sec and 60°C/1min. 5-HTR 7.1 mRNA abundance was quantified by the ΔΔcT method relative to GAPDH.

### Planarian mobility assays

Starved worms were exposed to drug / vehicle for five minutes, after which 10 animals were placed in drug-containing solution in the middle of a glass watchglass (50mm diameter, Fisher Scientific) centered over a LED backlit light (Edmund Optics, #83–873). Movement was captured using a digital video camera (Canon VIXIA HF R400) over a 2 minute period (30 frames per second). Representative images of this assay are displayed as minimal intensity z-projections (ImageJ) to provide a qualitative visual readout of experimental manipulations. The resulting videos were processed using custom written algorithms in C_trax_ to track the motility of individual worms [59]. Motion was scored by quantifying total distance travelled (mm) over the fixed recording interval and averaged for the 10 worms in each assay. Errors in tracking were corrected using the Fix Errors Matlab Toolbox and descriptive statistics were computed using scripts in the Behavioral Microarray Matlab Toolbox and custom written algorithms in MATLAB.

### Schistosomule isolation and contractility assays

*Biomphalaria glabrata* snails exposed to miracardia (NMRI Puerto Rican strain of *Schistosoma mansoni*) were obtained from the Biomedical Research Institute (Rockville, MD) and maintained at 26°C for 4 to 6 weeks. Isolation of matured cercaria and their transformation into schistosomules were performed as previously described [20]. For contractility assays, a custom written plugin (wrMTrck) in ImageJ was used to resolve schistosomule body length (major axis of an ellipse) over time following drug exposure (30min), as previously described [20]. For experiments on PZQ and 5-HT action on schistosomules, Basch media was made without 5-HT and drugs were added to the concentrations indicted.

## Supporting Information

S1 Text Fig A. Variation in S7 receptor transcripts during regeneration. Changes in FPKM values for individual S7 transcripts (7.1–7.8) at early regenerative timepoints during tail regeneration from an excised *D. japonica* head fragment. **Fig B. Sequence comparison with human GpCRs. Maximum likelihood cladogram (PhyML) of flatworm and mammalian GpCRs.** Sequence homology suggests closest resemblance of *D. japonica* (S1, S4, S7; colored) and *S. mansoni* (grey) sequences to serotonergic GpCRs (dashed lines) compared to GpCRs with other ligand specificities (adrenergic, dopaminergic, histaminergic, muscarinic). Analysis was performed based on protein sequence alignment (MUSCLE). Human sequences were retrieved from Uniprot by the following identifiers: 5HTR1A, P08908; 5HTR1B, P28222; 5HTR1D, P28221; 5HTR1E, P28566; 5HTR1F, P30939; 5HTR2A, P28223; 5HTR2B, P41595; 5HTR2C, P28335; 5HTR4, Q13639; 5HTR5A, P47898; 5HTR6, P50406; 5HTR7, P34969; HRH1, P35367; HRH2, P25021; HRH3, Q9Y5N1; HRH4, Q9H3N8; ACM1, P11229; ACM2, P08172; ACM3, P20309; ACM4, P08173; ACM5, P08912; DRD1, P21728; DRD5, P21918; DRD2, P14416; DRD3, P35462; DRD4, P21917; ADRA1A, P35348; ADRA1B, P35368; ADRA1D, P25100; ADRB1, P08588; ADRB2, P07550. *S. mansoni* predicted transcripts [24] were retrieved from GeneDB by the identifiers Smp_149770, Smp_197700, Smp_126730, Smp_148210. Analysis was bootstrapped with 500 replicates. **Table A. Diverse serotonergic antagonists cause bipolarity.** Structurally diverse ligands evidenced to act as mammalian serotonergic blockers produce 2-headed worms in the planarian trunk fragment regeneration assay. Maximal penetrance of the two-headed phenotype is scored (highest level of bipolarity at doses of drugs that are not toxic). Compounds sources: 1Sigma Aldrich, 2Tocris Bioscience. **Dataset A. Sequences used for RNAi for S7 receptors.** Amino acid sequences for S7 receptors (derived from the transcriptome assembly). Regions used for RNAi (bold) were amplified by PCR from planarian cDNA and cloned into the RNAi vector (see Methods). Attempts at PCR amplification of S7.8 (lowest predicted expression by FPKM values) were unsuccessful. **Dataset B. Sequences of S1 and S4 planarian 5-HT receptors.** Amino acid sequences for 5-HT receptors (derived from the transcriptome assembly).
(DOCX)
