# Supplementary material for: Ergot Alkaloids (Re)generate New Leads as Antiparasitics
Source: PLoS Negl Trop Dis. 2015 Sep 14;9(9):e0004063. doi: 10.1371/journal.pntd.0004063 (PMC4569474; doi:10.1371/journal.pntd.0004063)
Supplement: S1 Text — Changes in FPKM values for individual S7 transcripts (7.1–7.8) at early regenerative timepoints during tail regeneration from an excised D. japonica head fragment. Fig B. Sequence comparison with human GpCRs. Maximum likelihood cladogram (PhyML) of flatworm and mammalian GpCRs. Sequence homology suggests closest resemblance of D. japonica (S1, S4, S7; colored) and S. mansoni (grey) sequences to serotonergic GpCRs (dashed lines) compared to GpCRs with other ligand specificities (adrenergic, dopaminergic, histaminergic, muscarinic). Analysis was performed based on protein sequence alignment (MUSCLE). Human sequences were retrieved from Uniprot by the following identifiers: 5HTR1A, P08908; 5HTR1B, P28222; 5HTR1D, P28221; 5HTR1E, P28566; 5HTR1F, P30939; 5HTR2A, P28223; 5HTR2B, P41595; 5HTR2C, P28335; 5HTR4, Q13639; 5HTR5A, P47898; 5HTR6, P50406; 5HTR7, P34969; HRH1, P35367; HRH2, P25021; HRH3, Q9Y5N1; HRH4, Q9H3N8; ACM1, P11229; ACM2, P08172; ACM3, P20309; ACM4, P08173; ACM5, P08912; DRD1, P21728; DRD5, P21918; DRD2, P14416; DRD3, P35462; DRD4, P21917; ADRA1A, P35348; ADRA1B, P35368; ADRA1D, P25100; ADRB1, P08588; ADRB2, P07550. S. mansoni predicted transcripts [24] were retrieved from GeneDB by the identifiers Smp_149770, Smp_197700, Smp_126730, Smp_148210. Analysis was bootstrapped with 500 replicates. Table A. Diverse serotonergic antagonists cause bipolarity. Structurally diverse ligands evidenced to act as mammalian serotonergic blockers produce 2-headed worms in the planarian trunk fragment regeneration assay. Maximal penetrance of the two-headed phenotype is scored (highest level of bipolarity at doses of drugs that are not toxic). Compounds sources: 1Sigma Aldrich, 2Tocris Bioscience. Dataset A. Sequences used for RNAi for S7 receptors. Amino acid sequences for S7 receptors (derived from the transcriptome assembly). Regions used for RNAi (bold) were amplified by PCR from planarian cDNA and cloned into the RNAi vector (see Methods). Attempts at PCR amplification [file pntd.0004063.s001.docx]

**

**

**Figure A in S1 Text**

**
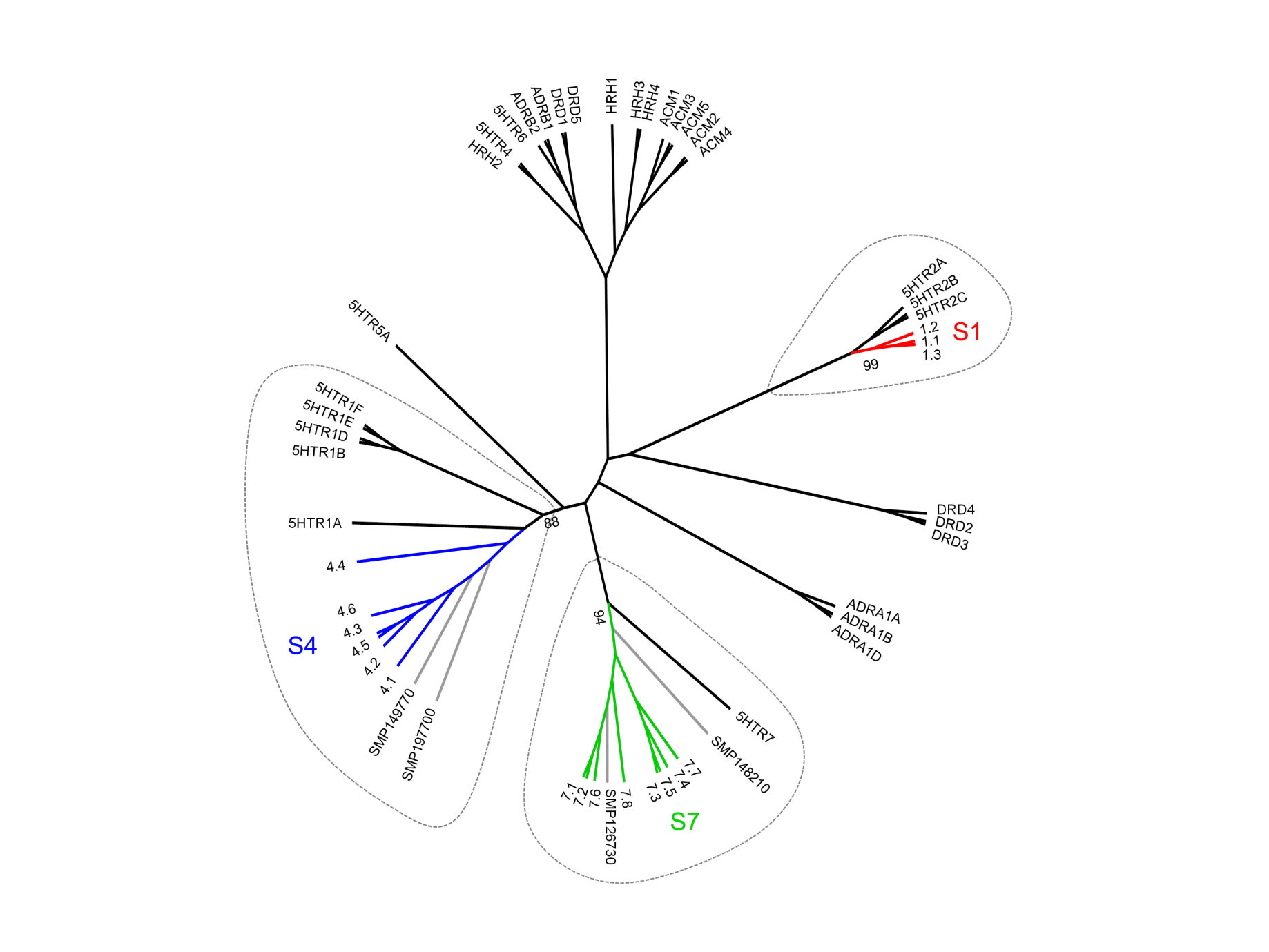
**

**Figure B in S1 Text**

| **Drug** | **Activity** | **Phenotype** | **Penetrance** | **Conditions** |
| --- | --- | --- | --- | --- |
| Dimebon | Antagonist | 2-head | 24±13% | 10µM, 48hrs ^1^ |
| DR 4485 | Antagonist | 2-head | 21±8% | 1µM, 48hrs ^2^ |
| Methiothepin | Antagonist | 2-head | 12±6% | 2.5µM, 24hrs ^1^ |
| Cinanserin | Antagonist | 2-head | 11±5% | 10µM, 48hrs ^2^ |
| Ketanserin | Antagonist | 2-head | 8±2% | 10µM, 48hrs ^1^ |
| Sertindole | Antagonist | 2-head | 3±1% | 1µM, 48hrs ^1^ |
| Spiperone | Antagonist | 2-head | 2±1% | 10µM, 24hrs ^1^ |

**Table A in S1 Text**

**Dataset A in S1 Text**

>S7.1

MWKLPFVFIIISNSF**SIVVMQDLILNCTNYSIYVTVNNLSRLAIPFECEKYPVGMEVFLGIIFFIIVAGAIGGNFLVILAIILVKKLQTASNWLILSLAFSDFFVSVLVMPIAAFNQLSRFRWPFSEKLCDFYNCCDVMLCTSSILNLCAISIDRYLVITRPMQYVVRRTPALIGGMIGVAWLMSGLISIPPVLGWKEKFTPGICQLTDNLLYQIYATFCAFYIPLIVMLVLYYQIFKLARNMAQEDAKRKLGTGQMTDEEQTSIPIQLGRTNSGDEDRKLLRLEDAQRLSKGNQGNGFDPEAQTGPKNNAKKKKQVNNESKAITTLGVIMGCFTLCWLPFFIIQILKPILIVSKVDHEKYLMPWCYELFLWLGYFNSFLNPVIYAKFNREFRNPFKQILFFHCFNINARIRVDTFAEQYGLPMQKTMATSLYETSHLNTHNTANSRRRSS**VPFTPVSRKNKSSSQSRPTSDQSTNKA

>S7.1

ATGTGGAAATTACCATTTGTTTTTATTATAATCTCAAATTCATTT**TCTATTGTTGTCATGCAGGATCTTATATTAAATTGTACGAATTATTCAATTTATGTTACAGTAAACAATCTATCAAGGTTAGCTATTCCATTCGAATGTGAAAAATATCCGGTTGGTATGGAAGTATTTCTGGGGATTATATTTTTTATTATCGTTGCGGGAGCTATCGGAGGTAACTTCTTAGTAATATTAGCTATTATTCTTGTAAAAAAATTGCAAACCGCAAGTAACTGGTTAATTTTGAGTTTGGCTTTCAGTGATTTTTTTGTTTCTGTTCTTGTTATGCCAATTGCTGCATTCAATCAATTGTCAAGATTCAGATGGCCATTTTCGGAAAAACTTTGTGATTTTTACAATTGTTGTGATGTGATGCTTTGTACATCAAGCATTCTTAATCTTTGTGCTATTTCTATTGACAGGTATTTAGTAATTACCAGACCAATGCAATATGTTGTTCGTCGTACGCCAGCTTTAATCGGAGGAATGATAGGAGTAGCTTGGTTAATGTCAGGATTAATAAGTATACCACCAGTTCTTGGATGGAAGGAAAAATTTACGCCTGGTATTTGTCAATTAACTGATAATTTATTATATCAGATTTATGCCACATTTTGTGCCTTCTACATTCCTTTGATAGTCATGCTGGTGCTATATTATCAAATATTTAAACTTGCCAGAAATATGGCCCAAGAAGATGCCAAGAGAAAATTGGGCACAGGTCAAATGACAGACGAAGAACAAACCTCTATTCCTATTCAATTGGGAAGAACAAATTCCGGAGATGAAGATAGAAAACTTCTTAGGCTTGAAGATGCACAGAGATTGAGTAAAGGGAATCAAGGTAACGGATTCGATCCTGAAGCACAAACGGGTCCTAAAAATAATGCTAAAAAAAAGAAACAAGTAAACAATGAATCTAAGGCTATAACAACTCTTGGAGTTATTATGGGCTGTTTCACTCTATGCTGGCTTCCATTTTTCATTATACAGATTCTCAAACCTATTCTCATTGTTTCTAAAGTTGATCATGAAAAGTATCTTATGCCTTGGTGTTACGAGTTGTTTCTGTGGCTTGGTTATTTCAACAGTTTTTTAAATCCCGTTATCTACGCCAAATTCAACAGAGAATTTCGAAATCCATTCAAACAAATTCTATTTTTCCATTGTTTCAATATCAATGCACGTATTCGTGTTGACACATTTGCCGAGCAATATGGTTTACCAATGCAGAAAACCATGGCAACATCATTGTATGAAACCTCTCATCTTAATACTCACAACACTGCCAACAGTCGTCGTCGGAGTTCA**GTACCTTTTACACCAGTGAGTAGAAAAAATAAATCTTCTAGCCAATCAAGACCAACTTCAGATCAATCAACAAATAAAGCTTAAAATTTGA

>S7.2

MSTANSTNCTFYFQLASKNNSAVQVPFYCIKFSFEFEVFLIIVLTIFLVGTAGGNLLVISSVAIVKKLQTPSNFLIVNLACSDFLVSI**LVLPGAVHQVIYRGQWPFSEILCDIFISFDVILCTSSILNLCAISIDRYLVITRPLQYAVNRTPARIGTMVAISWVTSALISIPPMFGLKETFIPGQCNYSDNLIYQIYATFGAFYIPLIVMLILYGRIFKLAREMAQNDAKLKIGISPNSSDKEQHSHLRIVDSHICNTPNGQMNLSLYSGSLCSISKSNNLSDSSQNKINLRNRVKLKSSTETKAITTLGVIMGCFTICWLPFFLIQISKPVLKVANVDSMNYFPIWLIELCLWLGYFNSFLNPIIYAKFNREFRTPFKQILLCKCNSINARLRSETFVQEYGLPNSSAPTKRG**SFDQTSLNNGSFTKNRRRRTPGVLKQNPHLNNCKIENSRKIYECSLINKTNKFPPESSVLLNSIANGNTKPVKKDTDDNPFHFSDQISPNCSINDQRSEKYVYDFGPASESVSIDPPPPINVSSYNLNMDY

>S7.2

ATGTCAACTGCCAATTCCACAAATTGCACGTTTTATTTCCAGCTAGCTTCAAAAAACAACAGTGCAGTTCAAGTGCCATTTTACTGTATTAAATTCTCCTTTGAATTTGAAGTATTTCTCATCATTGTTTTAACAATTTTTCTTGTTGGAACAGCTGGCGGAAATTTATTGGTTATAAGTTCAGTTGCAATTGTTAAAAAGCTACAAACTCCATCAAATTTCTTAATTGTGAACCTTGCTTGTAGCGACTTTCTCGTATCGATT**CTCGTATTGCCAGGAGCAGTTCATCAAGTAATCTATAGAGGTCAATGGCCGTTTTCAGAGATACTTTGTGATATATTTATTTCTTTTGATGTAATTCTGTGTACATCAAGTATATTAAATCTTTGCGCAATATCAATCGATAGATATTTAGTTATCACTCGACCTTTACAATATGCAGTAAATCGAACACCAGCAAGAATTGGTACAATGGTTGCAATAAGTTGGGTAACCTCTGCTCTAATTAGTATTCCTCCAATGTTTGGATTAAAAGAAACATTTATACCAGGACAATGTAATTATAGTGATAATCTTATTTATCAAATTTATGCAACATTTGGCGCATTTTACATACCGCTTATTGTTATGCTGATTCTTTATGGAAGAATATTTAAATTAGCGCGAGAAATGGCCCAAAACGATGCGAAATTAAAAATTGGGATTTCTCCAAATTCTTCTGATAAAGAACAACATTCTCATTTGAGAATTGTCGACAGTCATATATGTAATACTCCTAATGGACAAATGAATCTATCACTTTATTCTGGTTCATTATGCAGCATTTCAAAGTCAAATAATTTGTCAGACTCATCACAAAATAAGATCAATCTTAGAAATCGAGTTAAACTTAAATCGTCTACTGAAACAAAAGCCATAACAACTCTTGGTGTAATTATGGGATGTTTTACTATATGTTGGCTACCATTCTTTTTGATACAGATTTCAAAACCAGTATTGAAAGTAGCCAATGTAGATTCGATGAATTATTTTCCAATCTGGTTAATTGA**

**GCTGTGCCTATGGCTAGGATACTTCAACAGCTTTCTGAATCCAATAATCTATGCCAAATTTAATCGAGAGTTTAGAACGCCATTTAAACAGATTTTATTATGCAAATGTAATTCAATAAATGCAAGACTGAGAAGTGAGACCTTTGTTCAAGAATACGGTTTACCGAATTCATCAGCTCCGACAAAACGTGGA**AGTTTTGATCAGACCTCATTGAATAATGGAAGTTTCACAAAAAATAGAAGACGACGTACACCAGGAGTATTAAAACAAAATCCTCACTTAAATAATTGTAAAATAGAAAACAGTCGAAAGATTTATGAGTGCTCACTCATTAATAAAACAAATAAATTTCCACCAGAATCCTCTGTTTTATTAAATTCAATCGCGAATGGTAACACAAAACCGGTTAAAAAGGATAC

AGATGATAATCCATTTCATTTTAGTGACCAAATATCTCCTAATTGCTCAATAAATGACCAAAGATCAGAAAAATATGTTTATGATTTTGGTCCAGCATCTGAATCTGTGAGTATTGATCCACCACCTCCTATAAATGTATCTTCATATAACTTGAATATGGATTATTAA

>S7.3

MNKRNLSDILQRINSNEMIDLEIIIDYFGSTNKSIQYTCEKAFITWNMTGLHNLPPQCLPERYYPVTLIILYIMLSILFLGTVIGNLLMIFAFILVRRLRTPCNMLIINLAVTDLLVGVLVIPLASIYQIKGFWIFNDIICSLFILFDVLLCTSSILNLCAISVDRYFII**TQPFRYATHRTTKKMLLMIFVVWIASALISVPPLFGWSKPKIKYSCDYSENLGYQIYATFIAFYLPLIVMLVLYGRIFRLANTIYNQEQLGDKPNKELSNLPSGNILKNNSNTLIKVENNCNNYPKEFIAPTIQINFAETDDETNNKLKRKFHSGKKSTEAKAIKTLGVIMGCFTICWLPFFIVQLLNPVFLAFGIQARTPVTAIFFQIFYWLGYTNSFLNPIIYAIFNKEFRTPLKQIIKCRCRNIEDILRSENYFSQYGMTQLKSNKSIKDSETPINRRRSPQRIRYTSSTISKAPV**

>S7.3

ATGAATAAACGTAATTTATCAGACATCCTTCAAAGAATTAATTCAAATGAAATGATAGATTTGGAAATTATTATTGATTATTTCGGATCTACAAATAAATCCATTCAATATACTTGTGAAAAGGCATTTATTACATGGAACATGACTGGTTTACACAACTTACCACCACAATGTTTACCAGAAAGATACTATCCAGTAACATTGATAATTTTATATATTATGCTGTCAATACTTTTTCTTGGAACTGTAATTGGGAATTTATTAATGATTTTTGCTTTTATATTGGTACGTCGATTACGAACTCCATGTAACATGTTAATAATTAATTTGGCTGTAACAGATTTATTGGTTGGTGTTCTGGTTATTCCATTAGCTAGTATTTATCAAATCAAAGGTTTCTGGATTTTTAATGATATTATTTGTAGTTTATTTATCTTATTTGATGTTCTTCTTTGTACATCGTCGATATTAAACTTGTGTGCAATATCTGTCGATCGCTACTTTATCATT**ACTCAGCCGTTTCGTTATGCAACTCATAGAACAACAAAGAAAATGCTTTTAATGATTTTTGTCGTATGGATAGCTTCAGCATTAATCAGTGTTCCACCATTGTTTGGTTGGAGTAAACCAAAGATTAAATATTCATGTGATTACAGTGAAAATCTCGGTTACCAAATATATGCAACATTCATTGCATTCTATTTACCCTTGATTGTTATGCTGGTTTTATATGGACGTATTTTTAGATTGGCCAACACTATTTATAATCAAGAACAATTGGGAGATAAACCAAATAAAGAATTGTCTAATTTACCAAGTGGAAACATTTTAAAAAACAACAGTAACACTCTAATCAAAGTTGAAAATAATTGCAACAACTACCCTAAAGAATTTATTGCGCCAACAATACAAATAAATTTTGCAGAGACTGATGATGAAACAAACAATAAATTGAAAAGAAAATTCCATTCTGGAAAAAAGAGTACAGAAGCTAAAGCCATTAAAACTTTAGGAGTAATTATGGGCTGTTTTACTATCTGTTGGTTACCG**

**TTTTTCATCGTTCAGTTGTTAAATCCGGTATTTTTGGCTTTCGGAATACAAGCACGAACACCTGTGACAGCAATATTTTTTCAGATATTCTATTGGCTTGGATACACAAATAGCTTTTTAAATCCAATAATTTACGCAATTTTCAATAAAGAATTTAGAACTCCGTTGAAGCAAATAATTAAATGTCGATGTAGAAATATTGAAGATATTTTACGTTCGGAAAATTATTTTAGTCAATACGGAATGACACAATTAAAATCTAATAAATCAATCAAAGATTCTGAAACTCCAATAAATCGTCGTCGAAGTCCCCAAAGAATAAGATATACTTCATCAACTATTTCTAAAGCTCCTGTTTGA**

>S7.4

MMANISNYTNVQKYIFINELILGIVFCILILGTVIGNGLVILSVVLAKRLQTPNNILIVNLA**ISDMLVALLVLPLATIYQLKGYWMFNEIVCDFFIVCDVLLCTASILNLCAISIDRYLVITKPFQYITKRNNKLMVTMVGISWLASALISIPPLFGWKQKFELGQCIYSDSLGYQIYATFGAFYIPSIIMIVLYGRIYQIAKHSAAMDSHLKSIRGSICKDPMTIHSCNSSYKSSQSDIQSQTSFSGHISSARHNINSNIYRSCTTTDLRHSCQLTLPDKRLVRNSFPGITDCQNKSNRNSRSNSKHSTTIRVLLSASRDTLTKRWKEIIHSSKRSSQSEGKAIFTLGVIMGCFCICWMPFFIIQILTPIFKIIHGHNPSAKLFTIPQSVSELFLWLGYFNSFLNPIIYVKFNKEFRTPFKEILFCRCRKLNA**RIRSESYMRQIGEFRKISKLSTTIASGEERKQSWRYNSVNYTEKK

>S7.4

ATGATGGCCAATATTTCAAATTATACAAATGTTCAAAAATACATTTTCATTAATGAATTGATTTTGGGAATTGTGTTTTGTATTTTAATACTAGGAACTGTAATTGGCAATGGATTGGTTATTTTATCTGTTGTGTTAGCGAAACGTTTACAAACACCGAACAATATCTTAATTGTTAATTTAGCG**ATAAGTGACATGTTGGTGGCTCTTTTAGTTTTACCATTAGCGACAATTTATCAACTGAAAGGCTATTGGATGTTTAATGAAATTGTGTGTGATTTTTTCATTGTTTGTGATGTGTTATTATGTACTGCATCGATTTTAAATCTATGTGCGATTTCAATTGATAGATATTTGGTCATTACTAAACCGTTTCAATATATTACGAAAAGAAATAATAAATTAATGGTAACTATGGTTGGAATATCATGGTTGGCATCAGCTTTAATTAGTATACCACCGTTATTTGGTTGGAAACAGAAATTTGAACTCGGTCAATGCATTTATAGTGATAGTTTAGGATATCAAATTTACGCTACATTTGGAGCATTCTATATTCCATCGATAATTATGATAGTTCTTTATGGTAGGATTTATCAGATAGCCAAACACAGTGCAGCAATGGATTCACATTTGAAAAGTATAAGGGGCTCTATTTGCAAAGATCCAATGACAATTCACTCTTGTAATTCATCGTATAAAAGCTCACAAAGCGATATACAAAGTCAAACATCGTTTAGCGGACATATTTCATCAGCTAGACATAATATTAATTCAAACATTTATAGATCATGCACTACAACAGATCTTAGGCATTCTTGTCAACTAACTCTTCCCGATAAACGATTAGTTAGAAACTCATTCCCGGGAATTACTGATTGTCAAAATAAAAGTAATCGTAATTCACGAAGTAATAGTAAACACTCAACTACAATTCGAGTTCTACTTTCGGCATCAAGGGACACATTAACAAAACGATGGAAAGAAATAATCCATTCATCGAAAAGATCATCGCAAAGTGAAGGAAAAGCTATATTTACACTCGGTGTAATAATGGGCTGCTTTTGTATTTGTTGGATGCCATTTTTCATAATTCAAATTCTAACTCCAATTTTCAAAATCATTCATGGTCACAATCCAAGTGCTAAATTATTTACAATACCTCAAAGCGTCAGTGAATTATTTCTCTGGTTAGGTTATTTCAATAGTTTTCTCAATCCAATTATTTATGTCAAATTTAACAAAGAATTTCGAACTCCTTTCAAAGAAATTTTGTTTTGTCGATGCCGGAAATTGAATGCT**CGTATTCGATCGGAATCATACATGAGACAAATTGGTGAATTTCGGAAAATATCTAAACTCAGTACAACTATAGCTTCAGGAGAGGAAAGGAAACAATCTTGGAGATATAATTCAGTGAATTATACGGAGAAAAAATAG

>S7.5

MLILNLAVTDLLVGILVIPFASIYQIKGYWIFDEIVCDIFILFDVLLCTSSILNLCAISVDRYLVITQPF**KYAVKRTRKRMLCMILLVWTASGLISIPPLFGWRQKRSPYTCDYSEDLGYQIYATFIAFYLPLVVMLVLYGRIFRLASAMLAAELKNKINTDCSESITTSVSRRASFTDEGDDTNAKGTLQVVGNFFKPISRRLSFKITTDDSNTIYNPVFDEMGLSPQKNPFFNRRSSDISKTSYSVTNNRKRNQNTIDVPVIHIDYSGIEDEHTNRDFSPNKPTLNFHKPQKACVEAKAVRTLG**IIMGCFTVCWLPFFLMQILFPILKILKVDYGGTATNICFQTFYWLGYTNSFLNPLIYAKFNREFRRPFCQILMCHCRHINAHLRSERYNDQYGTNKTRQRSIQHRLSTTYSTLPPVASSRNGRMSRTRRSTSILSDCECTNLSPTKCTNQTHLLIP

>S7.5

ATGCTGATTCTTAATCTGGCTGTAACTGATTTATTAGTAGGTATACTAGTTATACCTTTCGCAAGTATTTATCAAATCAAAGGATATTGGATATTTGATGAGATTGTGTGTGATATTTTTATTCTTTTTGATGTCTTATTATGCACTTCATCTATACTTAATCTTTGTGCAATTTCCGTGGATCGATATCTTGTTATAACGCAACCTTTT**AAGTATGCTGTAAAACGGACACGAAAACGAATGCTATGCATGATTCTTTTAGTTTGGACAGCTTCTGGTCTTATTAGTATACCACCCTTATTTGGTTGGAGACAAAAAAGATCTCCGTATACATGTGATTATAGTGAAGATTTGGGATATCAAATTTATGCAACATTTATTGCATTCTATCTACCGTTGGTTGTAATGTTAGTGCTGTATGGTAGAATCTTTAGATTGGCTAGTGCAATGTTAGCTGCAGAATTGAAGAATAAAATAAACACTGATTGTTCAGAATCAATAACGACCAGTGTTTCCCGGCGTGCTTCATTTACTGATGAAGGTGATGATACTAATGCAAAAGGAACTTTACAAGTTGTCGGTAACTTTTTCAAACCAATTTCTCGACGTTTAAGTTTCAAAATTACTACCGACGATTCCAATACAATTTATAATCCTGTATTCGATGAAATGGGCTTAAGTCCACAGAAAAATCCATTTTTCAATAGAAGATCCTCAGATATTAGTAAAACTAGTTACAGTGTTACAAATAATAGAAAACGAAATCAAAATACAATTGATGTTCCTGTTATTCATATCGATTATTCAGGGATTGAAGATGAACATACTAATAGAGATTTTTCTCCAAATAAACCAACGCTTAATTTTCATAAACCACAAAAGGCATGTGTAGAGGCTAAAGCCGTGAGAACTTTGGGA**ATTATTATGGGATGCTTTACAGTTTGTTGGCTACCATTTTTTCTGATGCAAATTCTGTTTCCAATATTAAAAATACTTAAAGTGGATTATGGTGGGACTGCAACTAACATATGTTTCCAGACCTTTTATTGGTTAGGTTACACAAACAGCTTTCTCAATCCGCTAATTTATGCGAAATTTAATAGAGAATTTCGACGTCCATTTTGTCAAATTCTAATGTGCCATTGTCGACATATAAATGCTCATTTGAGATCAGAAAGATATAATGACCAATATGGTACAAATAAAACTCGCCAAAGATCGATACAGCATCGATTATCTACGACATATTCGACTTTACCTCCTGTGGCTTCTTCGAGAAATGGTCGAATGAGTCGAACAAGAAGATCTACATCAATTCTCAGTGATTGTGAATGTACCAATCTATCTCCAACAAAATGTACAAATCAGACTCATCTTCTTATTCCA

>S7.6

**MFNNSKFPVNCSTVVEDSDNTPLICLRYSSSIEQLLIVLFSLLIFGTLGGNCLVISAIILVKKLQIPSNFLILSLAASDTLVAFILPLVAFIQLRGGHWPFSETFCDIFICLDVLLCSSSILNLCAISIDRYLIITRPLQYGTRRTVKQMFIMILLVWFLSGLISIPPAIGWKERFIPGKCIYSENLGYQIYATCGAFYIPLIIMLVLYGRIFLLTQSMVKKDAQLSKRASRYLSSHLKMADLNDDSLVKENMCKRLSLSDQSSNEWIDSKKFTILQPKEEYIKDYRKTDKTNYAENKAITTLG**VIMGCFTICWLPFFVIQLIKPLLKVANIDSNLYISQWIQDMFLWLGYINSFLNPIIYAKFNREFRTPFKMILLCKCVGINAAIRSEAYAEQYGSRIPPCSSRRNKYIQKTFQKQTEVKYKNCNSKRNTLIEKV

>S7.6

**ATGTTCAATAACAGTAAATTTCCCGTCAATTGTTCAACGGTTGTAGAAGATTCTGATAATACACCATTGATTTGTTTGAGATACAGCTCATCAATTGAACAATTGTTAATTGTTTTATTTTCTCTATTGATTTTCGGAACTCTCGGAGGAAATTGTCTAGTAATTTCTGCAATTATTTTGGTGAAAAAACTTCAAATTCCCAGTAATTTCTTAATTTTGAGTTTAGCAGCCAGTGATACTTTAGTTGCGTTTATTCTACCATTAGTTGCTTTTATTCAGTTACGCGGTGGACATTGGCCGTTTTCAGAAACGTTTTGTGATATATTTATTTGTCTTGATGTACTATTGTGCAGTTCAAGTATTCTAAATTTATGTGCTATATCAATCGACAGATACTTAATCATAACCAGGCCGTTACAATATGGTACTCGAAGAACAGTAAAACAAATGTTCATTATGATTTTATTGGTTTGGTTTCTTTCTGGACTAATTAGTATTCCACCGGCTATCGGATGGAAAGAAAGATTTATTCCTGGAAAATGCATTTATTCAGAAAATCTTGGTTACCAAATCTATGCTACTTGTGGAGCATTTTATATCCCATTAATTATAATGCTCGTATTGTATGGACGAATTTTTTTATTGACCCAATCTATGGTGAAAAAAGACGCTCAACTAAGTAAACGAGCTTCCCGTTATTTATCATCCCATTTGAAAATGGCAGATTTAAATGATGACAGTTTAGTAAAGGAAAATATGTGTAAACGTTTATCGTTATCAGATCAAAGCAGTAACGAATGGATAGATTCGAAAAAATTCACGATTTTACAACCGAAAGAAGAATATATCAAAGACTACCGGAAAACTGATAAAACAAATTATGCAGAAAATAAAGCCATCACAACTCTCGGT**GTTATCATGGGATGTTTCACTATTTGTTGGTTACCGTTTTTTGTGATTCAATTGATCAAACCACTTTTAAAAGTTGCAAACATAGATAGTAATTTATACATTTCACAGTGGATTCAGGACATGTTTTTGTGGCTTGGCTACATCAATAGTTTTCTAAACCCGATTATATATGCCAAATTTAATAGAGAATTTCGAACTCCATTTAAAATGATTTTGTTATGTAAATGTGTTGGAATTAATGCTGCAATACGATCAGAGGCCTATGCTGAACAATACGGATCACGAATACCGCCCTGCAGTTCCAGGCGCAATAAATATATTCAAAAAACATTTCAGAAGCAAACTGAAGTGAAATATAAAAATTGCAATAGTAAAAGAAATACACTTATTGAGAAAGTTTGA

>S7.7

MNRSSVTQFGILIINNNNNYNYINNNNYSAFEICKSFYELLNNNSIGFSNLPQVCRKYTPTQEVIIGLILGVLTCATLIGNVLIIVSVTLVKKLQTPSNLLIVNLATSDLLVSGIVLPFAIIFQLQGFWPFSELLCDIFILSDVLLCTCSILSLCIISIDRYFAIT**RPLQYASKRTTGRISVMIVLVWLLAGLISVPPFIGWKEKFVKNECKYSDSLSYQIYATFGAFYVPLFIMLILYGKILLLAKRIANLDIKQRSLKTSRRNSAVVFSNQNANETRKPSLQPTMNCSFVQRDNGGLIDKPDELTFCKRYTITKLYNRTSMNLYKKSKLSSEVKAIKTLGIIMGCFTICWCPFFIIQLTIPIAKALGYEPNFVPPTV**IEIFLWLGYYNSFLNPIIYAKFNRDFRTPFKEILLFRCRTINRRMRSESYVEQYGMMNRRRSTMDVTYNNNARKLTKTREDFNYSLRFSRK

>S7.7

ATGAATCGTTCAAGCGTGACGCAATTTGGAATTCTCATAATTAATAACAATAATAATTATAATTATATTAATAATAATAATTATAGTGCATTCGAAATTTGTAAATCTTTCTATGAATTGCTGAACAACAATAGTATTGGATTTTCAAATCTACCTCAAGTTTGTAGAAAATACACACCAACTCAAGAGGTTATTATAGGATTAATATTAGGTGTTTTGACTTGTGCAACATTGATCGGTAACGTTCTAATTATTGTTTCTGTGACTCTCGTAAAAAAACTTCAGACTCCTAGCAATTTGCTAATTGTTAATCTTGCTACCAGTGATTTATTGGTGTCAGGTATTGTTTTGCCATTTGCAATCATTTTTCAATTACAAGGCTTCTGGCCATTTTCTGAATTATTATGTGATATTTTTATTTTATCAGATGTATTGTTGTGTACGTGCTCAATTCTTAGTTTATGTATCATATCTATCGATAGATATTTTGCCATTACT**CGACCACTGCAATACGCATCTAAACGAACAACCGGTCGAATCTCGGTGATGATTGTTTTAGTTTGGTTGTTGGCTGGTTTGATTAGTGTTCCGCCATTCATTGGTTGGAAAGAGAAATTCGTTAAAAACGAATGTAAATACAGTGATTCATTAAGCTATCAAATTTATGCTACATTCGGAGCATTCTATGTTCCGCTTTTTATCATGTTGATTTTATATGGGAAAATTCTATTATTAGCAAAACGAATAGCTAATCTTGATATCAAACAAAGAAGTTTAAAAACATCTCGTAGAAATTCAGCTGTTGTTTTTTCGAATCAAAATGCCAATGAAACAAGAAAACCGTCTTTACAACCGACAATGAACTGCTCATTTGTTCAACGGGATAATGGAGGATTGATTGATAAACCAGACGAGTTGACATTCTGTAAACGATATACAATAACGAAATTATACAATAGAACAAGCATGAATCTTTATAAAAAATCAAAGTTGTCATCAGAAGTTAAAGCGATTAAAACTCTCGGAATAATTATGGGATGTTTTACTATTTGCTGGTGTCCATTCTTTATAATACAGTTAACTATTCCCATTGCTAAAGCGTTGGGTTATGAACCGAATTTCGTACCGCCAACAGTC**ATTGAAATTTTTCTTTGGTTAGGATATTACAACAGTTTTCTAAATCCGATTATTTATGCAAAATTTAATCGAGATTTCCGAACCCCATTCAAAGAAATTTTGTTGTTTCGTTGTCGGACAATTAACAGACGAATGAGATCAGAATCATATGTGGAGCAATATGGAATGATGAATCGGCGCCGATCAACAATGGATGTAACATATAACAATAACGCAAGAAAACTAACTAAAACTCGAGAGGATTTCAATTATTCTCTTCGATTTTCAAGAAAATAA

>S7.8

MWKSPKQIFYLEVDEMNNSNSTTLNKSSLIERYSILSQITLATMLSIICVGTIIGNLLIIIAVYLVRKLHTPSNCLVFSLACSDFLVAIIVIPFAIITQIKGFWLFSELICDFFICLDILFCTASILNLCCISIDRYCTILQPLSRYGTDFRPVIIMICCIWILSGLISIPPVTGWKEQFHSGQCTYSESLWYQTYATLGAFYIPLIVMLILYSRIYQVARKIAKNNHMNNTFMGVKFSKHGPRDLIFKQFSLIASPLNSIPVVKNSLSPSNRYSQVLTSDNTYFKYHMANDIISVSTLQQRKSDESDMTNSDYWNSTKSKKAKQKYLSKKIMSESKAIKTLGILMGCFTFCWFPFFTLQLIVPVIKIYNINTKYIPIWLRETFLWLGYFNSFLNPIIYAKFNKDFRQPFKEILSCHCKSINSRLRTASYTEQFGKSIVQRRRSGCDSTPQLPVSRRSSSRPHKTPNHL

>S7.8

ATGTGGAAATCACCGAAACAAATTTTTTATTTGGAAGTTGATGAAATGAACAACTCGAATTCTACAACATTGAATAAATCGTCACTAATAGAAAGATATTCAATTTTATCCCAGATTACTTTAGCAACAATGCTGTCAATTATTTGCGTTGGAACAATTATTGGAAATTTGCTAATTATTATTGCAGTTTATTTAGTAAGAAAACTACACACGCCCAGCAATTGTTTAGTTTTTAGTCTAGCATGTAGTGATTTTCTTGTGGCAATAATCGTGATTCCGTTTGCAATAATAACACAAATCAAAGGTTTCTGGCTTTTTAGTGAGTTGATTTGTGATTTTTTCATTTGTCTGGATATTCTGTTTTGTACAGCATCTATTCTTAATTTATGTTGTATTTCCATCGATCGTTATTGTACCATTCTTCAACCTCTCAGCCGATATGGAACTGATTTTCGGCCGGTTATTATTATGATTTGTTGTATTTGGATACTTTCTGGACTTATTTCAATTCCACCTGTGACCGGTTGGAAAGAACAGTTTCACTCAGGTCAATGCACATACAGTGAAAGTTTATGGTATCAAACGTACGCTACATTAGGTGCATTTTATATACCTCTAATAGTTATGCTGATTCTTTACAGTCGAATCTATCAAGTGGCTAGAAAAATTGCCAAAAATAACCATATGAATAATACTTTTATGGGGGTAAAATTCTCAAAACACGGTCCACGAGATTTAATTTTTAAGCAGTTTTCTCTAATTGCATCTCCATTAAATTCAATTCCTGTTGTTAAGAATTCTCTCAGTCCTAGTAATCGTTACTCACAGGTACTAACTTCCGATAATACGTATTTTAAATATCACATGGCCAATGATATAATATCAGTTTCTACATTACAACAAAGAAAAAGCGATGAATCAGATATGACAAATTCAGATTATTGGAATTCTACAAAATCAAAAAAAGCAAAACAAAAATATCTTTCTAAGAAAATAATGTCTGAAAGTAAAGCTATAAAAACTCTTGGAATATTAATGGGATGTTTCACATTTTGTTGGTTTCCTTTTTTTACACTACAGTTAATAGTTCCGGTGATTAAAATATACAATATCAATACCAAATACATTCCTATTTGGCTCAGAGAAACCTTCTTATGGCTAGGATATTTTAACAGTTTTCTCAATCCAATAATTTACGCCAAATTTAATAAAGATTTCAGACAACCGTTTAAAGAAATTCTTTCTTGTCATTGTAAATCGATAAACAGTCGGTTAAGAACTGCTTCATACACAGAGCAATTTGGAAAATCGATTGTTCAAAGAAGACGTTCAGGTTGCGATAGTACTCCACAACTTCCTGTTTCTCGTCGGAGTAGCAGTCGACCACATAAAACACCGAATCATCTTTGA

**Dataset B in S1 Text**

>S4.1

MNISSTDNYTNNRNQSLEIARFSPKHGWIHVYLTSAILGFLILCTIIGNVFVVAAIILERNLQSVSNYLILSLAVADLMVASLVMSVSLLFEISKQWFLGIELCDFWVCFDVMCCSASILHLVAIAFDRYWAVTNIDYIRMRTAKRIIIMIVLVWLLSIIISIPSRFYSTRTWNKVYKSVVIDGRCDINDEYFYTIFSTVGAFYMPMFLLIGIYLKIYQTARARIRKHGIHKSNSKLNNNIVNSIKKCPFRWPRKVKFIRVKHELNSTHRYQCNSSYTQNSNYVQMKIDMQSYAISTFYNKNLSKTQVTSFDVDSINPSSCEPQSSSSSHKFCNSSSEHINKNQNHQITNSNIHSNNTAFNHYQCKHEKANCLFSTLQSPVVLESEFVNETVNHPDKYVEKVVWNWKDVSSLNNSPRLTCPNRPRHDCVEYLKKTKRKFYRHNNHNALSPNIYYKITGLLVPDRINFNKKKYSLTNRNNSIAVNINRRISNNINQIGTQSSRSDTYPNALVTNSDFTSRRTFVEQQKQKLEHKRERKAARTLAIITGCFILCWLPFFIQALILPFCGENCVQNEVVSSFFLWLGYCNSLLNPIIYTIFSPEFRNAFNKILFGRYQKTKRSY

>S4.1

ATGAACATTTCTTCAACTGATAATTATACAAATAATAGAAACCAATCATTGGAAATCGCAAGATTTTCTCCTAAACATGGATGGATTCATGTTTATTTAACATCTGCCATTCTCGGTTTTCTTATACTTTGTACTATAATCGGAAATGTATTTGTTGTTGCAGCGATAATCTTGGAACGTAATTTACAAAGCGTATCAAATTATCTTATTCTCAGCCTTGCTGTTGCAGATCTGATGGTAGCTTCCTTGGTTATGTCAGTCAGTCTTTTATTTGAGATTTCAAAACAGTGGTTTTTAGGTATAGAACTCTGTGATTTCTGGGTATGCTTTGATGTAATGTGTTGTTCTGCATCAATTCTTCACCTGGTTGCGATAGCATTCGACCGGTATTGGGCTGTAACGAATATTGATTATATTCGAATGAGGACAGCTAAACGTATAATAATCATGATTGTTCTCGTCTGGTTACTTTCTATTATCATATCGATTCCGTCTCGTTTCTATTCAACTCGAACATGGAATAAAGTCTATAAAAGTGTTGTTATAGATGGACGATGTGACATAAATGATGAATATTTCTATACAATATTTTCTACTGTTGGTGCTTTCTATATGCCAATGTTTCTTTTG

ATTGGTATTTATTTAAAGATTTATCAGACAGCCCGAGCGAGAATTCGCAAACATGGTATACATAAATCCAATTCAAAACTAAACAATAATATCGTAAACAGTATTAAAAAATGTCCATTTCGATGGCCGAGAAAAGTAAAATTTATCCGAGTTAAACATGAACTCAATAGTACTCATCGATATCAGTGCAATTCCAGTTATACTCAAAATAGTAATTACGTACAAATGAAAATAGATATGCAATCGTATGCAATTTCAACATTCTATAATAAAAATCTTTCTAAAACTCAGGTTACCTCATTTGATGTTGATTCAATAAATCCAAGTTCATGTGAACCCCAATCTAGTTCATCATCACATAAATTTTGCAATTCTAGTAGTGAACATATTAATAAAAATCAAAATCATCAAATTACAAAT

TCAAATATCCACTCAAATAATACAGCATTCAATCATTATCAATGTAAACATGAAAAGGCCAATTGTCTATTTTCTACTTTACAATCACCAGTAGTATTGGAATCGGAATTTGTGAATGAAACAGTAAATCATCCTGATAAATATGTGGAAAAGGTCGTATGGAATTGGAAAGATGTTAGCAGTCTCAATAATTCTCCAAGACTCACTTGTCCAAATCGTCCGAGACATGATTGTGTTGAATATCTAAAAAAAACCAAAAGAAAGTTTTATCGACATAATAATCACAATGCATTGAGCCCAAATATTTATTACAAAATAACTGGTTTACTAGTACCAGACAGAATAAATTTCAATAAGAAAAAGTATTCTTTAACTAACAGAAATAACAGTATTGCAGTAAATATTAATCGTAGAATTAGT

AACAACATTAATCAAATTGGTACTCAGTCTTCTCGATCAGACACATATCCAAACGCTCTTGTAACAAATAGCGATTTTACCTCAAGACGAACTTTTGTTGAACAACAAAAACAAAAACTCGAACATAAACGAGAACGTAAAGCTGCTCGAACACTCGCCATAATTACAGGGTGCTTTATTCTTTGCTGGTTGCCATTTTTTATACAGGCATTAATACTGCCTTTTTGTGGAGAAAATTGTGTTCAGAATGAAGTTGTCAGTTCTTTTTTTCTATGGCTTGGATATTGTAATTCTCTATTGAATCCAATCATTTATACAATTTTCTCTCCAGAATTCAGAAATGCATTTAATAAAATTCTTTTCGGTCGATATCAGAAAACCAAAAGAAGCTACTAG

>S4.2

ISSENAMSYGHQDEIVARSISSREEKISLRVNANAHMCTKEPACRQSLANNKNRLSNAMWHQLKIRSRLKIEQRRERKAARTLAIITGCFLICWLPFATQNMINLFSESANNTDHRSYKVLLIDSFILWLGYTNSCLNPIIYTVFSPDFRRSFRKILFGRYYHRGKRSQ

>S4.2

ATCAGCTCTGAAAACGCAATGTCGTACGGCCATCAAGATGAAATTGTAGCTCGAAGTATTTCAAGTAGAGAAGAAAAGATAAGTTTACGGGTAAATGCCAATGCCCATATGTGTACAAAAGAACCAGCATGCCGACAATCGCTTGCTAATAATAAAAATCGTCTTTCGAATGCAATGTGGCATCAACTTAAAATTCGATCTCGTCTAAAAATCGAACAAAGACGTGAAAGAAAAGCTGCTCGAACTCTAGCGATAATTACCGGTTGTTTTCTAATTTGTTGGCTACCATTTGCAACTCAAAATATGATCAATCTATTTTCAGAATCAGCAAATAACACAGACCATCGCAGTTACAAAGTTCTTTTAATAGACAGTTTTATATTATGGTTAGGTTATACTAATTCGTGCCTGAATCCTATA

ATTTACACGGTATTTTCCCCAGATTTTCGACGTTCTTTTAGAAAGATTCTATTTGGTCGTTACTATCATCGTGGTAAAAGATCGCAATAA

>S4.3

MNRVVNLTQPIQYEPRFQIWTMTFISLLLIIIIVWTIAGNVLVMVTIVTELNIKNSSNHLIFSLAVTDLFVAVTVMPLSAINEVSINWWLGLELCDIWIFTDVLTCTASIFHLVVIAIDRYLSITRLTYNRNRSMISTSAMIASSWIAAALVSIPSRFYLRRNNKIERLIVNYNGTCLINEEKPYIILANFLSFCFPLLIMGVVYFKIFIFIKKQTKTRKAIKNSVIGSDALSTTLASDICYSKSTVSPFPHSEIWSSIYEDSKIIPDENSFESIITESLDKSKFLNEECSTFQYADEEDECQSKLNINFGENISSKFVYCVDKSSTDSFQVSSVYLQPFTMKTYSEIFKPTNLAKTETMSTINGKVFPLDYQNNDILKSPISNKSIIKPSTSNSRKLKSPTLSWSPKLTFFPYRTSKSNMKPGFSFEETNYSLLTSTSPVKSNNNNHSLIGQLHSQISDNNNDNSFASMAIRVKRINQKREYKVVRTLFIITGFFVICWLPFGINNLLRAYVIINRSEIGRIMDSIFLWLGYANSGLNPIIYTIFSTDFQVAFKKILFGSNIS

>S4.3

ATGAACAGGGTAGTAAATCTAACCCAACCTATTCAGTATGAGCCCCGTTTTCAAATATGGACCATGACATTCATTAGTTTATTATTAATAATAATTATTGTTTGGACAATTGCTGGAAATGTACTGGTTATGGTTACTATTGTTACTGAACTAAATATTAAGAATTCTTCCAATCATTTGATTTTCAGTCTTGCAGTAACTGACCTATTTGTTGCTGTCACTGTTATGCCTCTGAGTGCTATAAACGAAGTTTCAATTAATTGGTGGTTAGGGTTAGAATTATGCGATATATGGATATTTACTGATGTTCTTACATGCACTGCATCAATATTCCATCTTGTAGTCATAGCAATAGATCGGTATTTATCTATAACAAGATTAACTTATAACAGAAATCGATCAATGATTTCTACAAGTGCTATGATTGCATCATCCTGGATTGCAGCTGCTCTTGTTTCCATTCCATCTAGATTTTATCTACGACGAAATAACAAAATTGAAAGATTAATTGTCAATTATAATGGGACCTGCCTCATAAACGAGGAAAAACCTTATATTATTCTAGCAAATTTTTTATCTTTTTGTTTTCCTCTTTTAATAATGGGTGTTGTTTATTTTAAGATTTTTATTTTTATAAAAAAGCAAACAAAAACCAGAAAAGCAATAAAAAATTCTGTAATCGGATCGGATGCTCTTTCGACAACATTGGCATCAGATATTTGCTATTCGAAATCAACAGTTTCTCCTTTTCCACATTCGGAAATTTGGTCATCGATTTATGAGGATTCTAAAATTATTCCAGATGAAAATTCATTTGAAAGTATAATAACAGAATCTTTGGACAAATCAAAATTTCTTAATGAAGAATGTTCAACTTTTCAATATGCCGACGAAGAGGATGAATGTCAGTCGAAATTAAATATAAATTTTGGGGAAAATATCTCGTCAAAATTTGTATATTGTGTAGATAAATCATCTACAGACAGCTTTCAAGTGTCTTCAGTTTATTTACAACCATTTACAATGAAAACGTACTCTGAAATATTCAAACCAACTAATCTAGCAAAAACAGAAACGATGAGTACAATCAATGGAAAGGTTTTCCCATTAGATTATCAAAACAATGATATTTTAAAATCACCAATCTCTAACAAATCTATTATAAAACCGTCAACGTCAAATTCAAGAAAACTAAAGTCACCGACTTTGTCATGGTCACCGAAATTAACTTTTTTTCCATATCGAACATCAAAGTCAAATATGAAACCCGGATTCAGTTTTGAAGAAACCAATTATTCACTTTTAACTTCAACGAGTCCTGTTAAATCCAATAATAACAATCATTCGCTAATAGGCCAGTTACATTCACAAATTTCTGATAACAACAACGACAATTCTTTTGCGTCAATGGCCATTCGCGTGAAACGAATTAATCAAAAACGTGAATATAAAGTCGTACGAACATTATTTATCATTACAGGTTTCTTTGTAATTTGCTGGCTACCATTTGGAATTAATAATTTACTACGAGCTTACGTTATTATTAATCGTTCGGAAATTGGAAGAATAATGGATAGTATATTTTTATGGTTGGGTTATGCAAATTCTGGATTGAACCCAATTATTTACACAATATTTTCAACTGATTTTCAGGTAGCATTCAAAAAGATCTTGTTTGGTAG

>S4.4

MVFNQTNFTQNFSVSTGFHLRYGKFHLVLTLFILETFIIMTIVGNILVILAVLLEKNLRNLSNYLILSMAVTDLIVALFVMNLSAINEISARWFLGIGWCDFWIFTDVLSCSASIFHLVTIAIDRYWSVTKIQYRHNKTMLHLIFTIISIWIISILIAIPSRFHFHRQKELMFNEVIIKGKCLINGGYFYTLISTFIAFYAPMVFMMIIYWKIYQVAKRQIRRKRKLNYPISINSKCKAICNYFHCCERSDDFEYSTNGEQYLITSSTKSKWNYSVEDETFSDFKYADNISVNSLNSEIKYKLNLKDVHIARMSSMSSLRTINVAIKPHNLTKNLDEQKRKLEIQRERKAIVTVGIITCCFILCWMPFFTLALVLPFCGNNCTIHPIISSVLLWLGYLNCLLNPFIYTFFSQEFRSAFKNILTGHYWARCIDL

>S4.4

ATGGTCTTCAATCAGACCAATTTTACTCAAAATTTTAGTGTTTCAACTGGATTCCATTTGAGATACGGTAAATTTCACTTGGTCTTAACATTATTCATTTTGGAAACTTTTATTATCATGACAATTGTCGGAAATATTCTTGTCATATTGGCCGTTCTTTTGGAAAAAAATTTGAGAAATTTATCCAATTATCTGATTCTCAGCATGGCTGTCACGGATTTAATTGTAGCATTATTTGTGATGAATTTAAGTGCTATTAACGAAATATCCGCTCGTTGGTTTTTAGGTATTGGTTGGTGTGATTTCTGGATATTTACAGATGTACTCAGTTGTTCGGCTTCCATTTTCCATTTGGTAACGATCGCAATTGATCGCTATTGGTCCGTAACTAAAATTCAATATAGGCACAACAAAACAATGTTGCATTTAATTTTTACAATAATTTCTATTTGGATAATTTCTATATTAATTGCAATTCCGTCGAGATTCCATTTTCATAGACAAAAGGAACTAATGTTTAATGAAGTCATTATTAAAGGAAAATGTTTAATTAATGGTGGCTACTTTTATACTCTAATATCAACTTTTATTGCTTTTTACGCTCCAATGGTTTTCATGATGATAATCTATTGGAAAATTTACCAAGTAGCTAAACGACAGATAAGACGGAAACGGAAATTAAATTATCCGATTTCAATAAACTCAAAGTGTAAAGCGATTTGTAATTATTTTCATTGTTGTGAAAGATCTGACGATTTTGAATACTCAACAAATGGTGAACAATATTTAATTACAAGTAGTACTAAAAGTAAATGGAATTATAGTGTAGAAGATGAAACCTTTAGTGACTTTAAATATGCTGACAACATATCTGTAAATTCATTAAATTCCGAAATTAAATACAAATTGAATCTTAAAGATGTTCATATAGCAAGAATGAGCAGTATGTCTTCTCTGAGAACTATCAATGTTGCGATAAAACCACACAATCTCACGAAAAATCTCGATGAACAAAAACGTAAATTAGAAATACAACGAGAAAGAAAAGCTATTGTGACTGTTGGGATTATTACGTGCTGTTTTATTCTTTGTTGGATGCCGTTTTTTACTTTAGCCTTAGTTTTACCATTTTGTGGTAACAATTGCACAATCCATCCAATTATTTCTTCAGTTTTATTATGGTTAGGTTATTTGAATTGTCTTCTTAATCCCTTTATCTACACATTCTTTTCTCAAGAATTTCGAAGTGCTTTTAAAAACATTTTGACTGGTCATTATTGGGCCAGATGTATTGATCTATAA

>S4.5

MVFYYPFENVTESQIARFSPRLSWIQFYITAILLGAVILWTIIGNVFVVAAIVLERTLKNVSNYLILSLAVADLMVAILVMPISAIKEVSSTWWLGIELCDLWTCFDVLSCTASILHLVVIAIDRYWAVTDINYLHKRTSSRIILMIIGVWLISVIISVPSRFHTHRNNAEWLRVIVNGSCIINESVSYTVFSNLGAFYLPMAFMSVIYLRIYIFARSRIRYKRKEMKQNRCLSNTDHKSCSNQTKPQEIIEQCPCDRRNIDEIGYIISCENCKLNSLVIQNQNRKEWFDKHMSLLEYADEEENDELSVHSNFFGTTFSNVKFESIDRVINISCIGENEKSKTEINSNNSIKKCNQTSNWSPKLIFFNYKTKLLNLKNDLSFKSSNRSTDLQFNDDRSQSSLSFKDNNKFNAMNSASIRNKIEQKRERKAARTLAIITGCFIICWLPFGLNNVAHAFCNTEPCGSALFDSLCLWLGYVNSGLNPVIYTVFSPDFRTAFRKILLGHYHTRRCFKC*

>S4.5

ATGGTGTTTTATTATCCATTTGAAAATGTTACCGAATCACAAATCGCTCGATTTTCACCCCGTTTATCATGGATACAATTTTATATTACAGCTATATTGTTGGGCGCTGTTATTTTATGGACTATTATCGGTAATGTTTTTGTAGTTGCAGCTATTGTTTTAGAGAGAACATTAAAAAATGTATCAAATTATTTGATTTTAAGCCTTGCAGTTGCTGATCTTATGGTGGCTATTCTTGTCATGCCAATTAGTGCAATTAAAGAGGTATCTAGTACTTGGTGGTTAGGAATTGAATTATGCGATTTATGGACATGCTTTGATGTATTGAGCTGTACAGCATCTATACTTCATTTAGTGGTTATAGCTATCGATAGATATTGGGCTGTAACAGACATCAATTACCTTCATAAACGAACGTCTAGCAGAATTATTCTAATGATTATCGGCGTTTGGCTTATTTCGGTTATAATATCAGTTCCTTCACGATTTCACACTCACAGAAACAATGCTGAATGGCTAAGAGTGATAGTTAATGGAAGTTGTATTATTAATGAATCTGTCAGTTACACAGTATTCTCCAATTTAGGAGCTTTCTATTTACCAATGGCATTTATGTCTGTAATATATTTAAGAATTTACATTTTTGCGAGATCTAGAATTCGATATAAACGAAAAGAAATGAAGCAAAATCGTTGCCTTTCAAATACCGATCATAAATCATGTTCTAATCAAACGAAACCACAAGAAATTATTGAACAGTGTCCTTGTGATCGTCGAAACATCGATGAGATCGGTTACATAATTAGTTGTGAAAATTGTAAACTTAATTCTTTGGTGATTCAAAATCAAAATCGAAAAGAATGGTTCGATAAACATATGAGTCTTTTAGAATATGCCGACGAAGAAGAAAATGATGAATTATCAGTTCATAGTAATTTTTTTGGAACAACATTTTCCAATGTGAAATTCGAAAGCATCGATCGGGTTATCAATATCAGTTGTATTGGAGAAAATGAAAAATCTAAAACAGAAATCAATTCGAATAATTCCATAAAGAAGTGTAATCAAACTTCGAATTGGTCACCGAAATTAATATTTTTTAATTATAAAACGAAACTATTAAATCTTAAAAATGATTTATCATTTAAAAGTTCAAATAGATCAACAGATTTACAATTCAATGATGACAGAAGCCAATCGTCGTTAAGTTTTAAAGATAATAATAAATTCAATGCAATGAATTCTGCTTCCATAAGAAATAAAATCGAACAAAAACGCGAAAGAAAAGCAGCTAGAACTTTAGCAATTATAACCGGATGTTTTATCATTTGTTGGCTGCCATTTGGACTTAATAATGTTGCGCATGCATTTTGTAATACAGAACCATGTGGAAGTGCTTTATTTGACAGTTTATGCTTGTGGCTTGGTTATGTTAATTCAGGCCTTAATCCAGTTATTTACACTGTTTTCTCTCCAGATTTTAGAACGGCTTTCAGGAAGATTTTATTGGGCCATTATCACACTAGACGATGTTTTAAATGTTGA

>S4.6

ILLEKNLQNVSNYLIISLAVADLMVASLVMSLSVFHEISAAWFLGLEICDLWICFDVLCCSASILHLVAIAIDRYMAVTKADYVRTRTARRIYIMIAMIWLLSIMISVPTRFHRNRNYFDEVQRVVYNGSCIINDDIGFTVFSTFGAFYLPMLFIIGIYFRIYQVARARIRRQAFRKRLRSQRNISKRLQPCICKSGYVNSKSETHLDCRNNKEINVYNIDEKTSINSCNNSSDSDSCCFIEKLNNSSIDLTCNQDSTITYMLNDTLSVSDDHKIINSNNRRLLNFECKTIPLNQFKKYLMKKSKALKVNKDSNKSRFVKSSTKTASVNNISTYEHRPSRMMTPSERLEYKRQKLEQKRERKAVRTLAIITVCFIVCWQPFFIQATIVPFCGKKCQLPPLLSSFVLWLGYCNSLLNPVIYTIFSPDFRDAFHKILFGRYLKNR

>S4.6

ATTTTGTTGGAAAAAAATCTTCAGAATGTATCCAATTATTTGATAATCAGTCTAGCAGTTGCTGATTTAATGGTGGCTAGTTTGGTTATGTCACTTAGTGTCTTCCATGAGATTTCAGCAGCCTGGTTCTTAGGTCTAGAAATTTGTGATTTATGGATTTGTTTTGATGTATTATGTTGTTCAGCTTCAATTCTTCATCTTGTAGCTATAGCTATTGATCGTTATATGGCTGTAACAAAAGCTGATTATGTGAGAACAAGAACAGCAAGAAGAATATACATAATGATTGCAATGATATGGTTGTTATCAATTATGATTTCAGTTCCCACAAGATTCCATCGTAATCGTAATTATTTCGATGAAGTACAAAGAGTTGTATACAATGGCAGTTGTATAATCAACGATGATATTGGATTTACTGTGTTCTCTACTTTTGGAGCTTTCTATTTACCGATGCTTTTTATAATTGGAATATACTTTAGAATATATCAGGTTGCACGAGCTAGAATTAGACGGCAAGCTTTTAGAAAACGCTTAAGAAGTCAACGCAATATTTCAAAACGTCTGCAACCATGCATATGCAAGTCTGGTTACGTAAATTCTAAATCAGAAACACATTTAGATTGTAGAAACAATAAAGAAATAAATGTTTACAATATTGATGAGAAAACATCTATAAATTCTTGTAATAACAGTTCGGATTCCGATAGTTGTTGTTTTATTGAAAAGCTGAATAATTCCTCGATAGATCTCACATGCAATCAAGATTCAACAATTACTTACATGTTAAATGATACACTTTCTGTTTCAGACGACCATAAAATTATAAACTCAAACAATCGAAGATTATTGAATTTCGAATGTAAAACAATACCATTGAATCAATTTAAAAAATATCTCATGAAAAAATCGAAAGCTTTGAAAGTAAATAAAGATTCAAACAAATCGAGATTTGTCAAATCTTCAACAAAAACTGCCTCAGTAAACAATATATCAACCTATGAACATCGGCCATCGAGAATGATGACTCCGTCAGAAAGATTGGAATACAAACGACAGAAATTAGAACAGAAACGAGAAAGAAAAGCTGTACGAACGCTTGCAATCATAACTGTTTGTTTTATTGTTTGTTGGCAACCGTTTTTTATTCAAGCTACAATTGTTCCATTTTGTGGTAAAAAATGCCAACTTCCACCTTTATTAAGTTCTTTCGTTTTGTGGCTTGGTTACTGTAATTCTCTACTAAATCCAGTGATTTACACAATATTTTCTCCAGATTTTCGAGATGCTTTTCATAAAATTTTATTTGGACGCTATTTAAAAAATAGATAA

>S1.1

LKERKFFPQSTFSLIKMNSEDNDESDNKNHSKCLNLFHSYDEENNYNNKLNNLYRPRSSIDFNSDNISSTYFPIINDQRFSDQNLPKQRFLSNIMYLKVENIRPQLARTVSFDETRELDRQELLKLPNDIENHSLENTDNNNELSAETITVQVDKNQTVISQDSLSLNYRQPNYLTPFLKKPETSSVEESTINCLLKHPLKLTNKMNLKNLPLLSITNRRNQRFHNHTKRNIRKSRKAVQVLGTLFGLFLLCYLPFFLVYIFDFFCSNCNGSLKDVIRHLEWVGYSASMLNPVVYHIFNPIFKMTFRRLLHGNCHKSRNNPHNISSFI

>S1.1

AACTAAAAGAAAGAAAATTCTTTCCCCAGTCAACATTTTCTCTAATTAAAATGAATTCAGAAGATAATGATGAAAGTGATAATAAAAATCACTCAAAATGTCTGAATCTATTTCATAGTTATGATGAAGAAAATAACTATAATAATAAATTAAATAATCTTTATCGGCCAAGATCTTCCATTGATTTTAATAGTGATAATATTAGTTCAACTTATTTCCCCATAATAAATGATCAAAGATTTTCCGATCAGAATTTACCAAAACAACGTTTCCTGTCAAATATCATGTATTTAAAAGTCGAAAATATTCGACCACAACTTGCTCGAACGGTAAGTTTTGATGAAACAAGAGAACTCGACCGACAGGAATTATTAAAACTTCCAAATGATATTGAAAATCATTCTTTAGAAAACACGGATAATAATAATGAATTAAGTGCTGAAACGATTACAGTACAAGTTGATAAAAATCAAACAGTAATATCCCAAGATTCATTATCATTGAATTACCGACAACCGAATTATTTAACGCCTTTTCTAAAGAAACCAGAAACGTCTAGTGTTGAAGAGTCGACAATTAATTGTTTATTGAAACATCCATTAAAATTGACAAATAAAATGAATCTAAAGAATTTACCGCTCTTATCAATTACAAACCGACGAAATCAAAGATTTCATAATCACACGAAAAGAAATATCAGAAAAAGTCGGAAAGCTGTCCAAGTATTGGGAACACTTTTCGGTTTGTTTTTATTATGTTATCTGCCATTTTTTTTGGTGTATATTTTTGATTTTTTCTGTTCAAACTGTAACGGATCATTAAAAGATGTAATTCGACATTTAGAGTGGGTTGGTTATTCAGCTTCTATGTTAAATCCGGTTGTTTATCATATTTTTAATCCAATTTTCAAAATGACATTTCGACGATTATTACATGGCAATTGTCACAAATCTCGAAATAATCCGCACAATATAAGCAGTTTTATTTAATGGTGATTACTGTGAAAATCTAGTTTTAAATTTTGAATTAATTGCTTATTTTTAATGAAAAACCTTTGAAAAAT

>S1.2

PRGICLAWYSLDVFFTSTTIIHLCSISLDRYFVLNNPLKYHNTRIRSSLPLKISIAWLIPFCIACPLFIFALHLDNNIQNPFIEMGQLSNQTMPPQMDSYKGCGPHNVYFIIIATVTTFVLPLCIMIVTYILTVWSIIRQTNQARQNLYPDKTVTKSRPHTHIKITDDSQSINETTNSAENVSYSLIGVDEKYCSHSSLSINSFDSPTKRRSFKISNKKEFKIHLNVPTMEIRRSFDFSPKSTVGIKKQSSLSEKSFNSFNNSFKMSPSRYTNQSSIRKNLSNSFRIHKQSRNSAKRTLAAMNSGKKAVQVLGILFGLFLLCYLPFFVIYLLDFFCTRCQSMTGPLVSKSEWIGYSASMLNPIVYHIFNPTFRNTFNRLMRCKCYKVRKAFSTNIKSYV

>S1.2

CCCAGAGGAATCTGCCTGGCTTGGTACAGTCTCGATGTGTTTTTTACATCCACTACGATCATTCATCTTTGTTCAATTTCGCTAGATCGATATTTTGTGCTTAACAACCCATTGAAATATCATAATACTCGAATCAGATCATCTCTGCCTTTAAAGATCAGCATAGCTTGGTTAATTCCATTTTGTATTGCATGCCCATTGTTTATATTTGCTCTTCATCTTGATAATAATATTCAGAATCCCTTTATCGAAATGGGTCAGTTATCCAATCAAACAATGCCACCACAGATGGATTCTTACAAAGGATGTGGACCACACAACGTTTACTTTATCATAATTGCTACTGTAACCACTTTTGTTTTACCACTCTGCATAATGATCGTAACTTACATCCTTACAGTGTGGTCCATAATTCGGCAAACTAATCAAGCACGGCAAAATCTATATCCCGACAAAACCGTAACAAAATCTCGCCCTCATACTCATATTAAAATTACTGACGACAGCCAGTCTATTAATGAGACAACAAACAGCGCAGAAAATGTTTCATATTCATTAATCGGAGTTGATGAGAAATACTGCAGTCATTCATCATTAAGTATAAATTCATTTGATTCTCCAACTAAAAGACGTTCATTTAAAATATCCAATAAAAAGGAATTCAAAATCCACCTCAATGTTCCCACCATGGAAATCAGAAGAAGTTTTGATTTCTCTCCAAAGTCAACTGTTGGGATCAAAAAGCAGTCGTCTTTATCAGAGAAAAGTTTCAACAGTTTCAATAATTCATTTAAAATGAGTCCATCTCGTTATACTAATCAATCATCGATTAGAAAGAACTTATCAAATTCATTTAGAATTCATAAACAATCAAGAAATAGTGCCAAAAGAACATTAGCTGCTATGAATTCCGGAAAGAAAGCTGTTCAAGTTCTTGGAATTCTGTTTGGTCTTTTCTTGTTATGTTATTTACCATTTTTTGTAATTTATCTGCTTGATTTTTTCTGTACGCGATGTCAAAGTATGACAGGTCCGTTGGTTTCTAAATCCGAATGGATTGGCTACTCAGCCTCTATGCTGAATCCTATTGTTTATCATATATTTAATCCGACATTCAGAAATACTTTCAACCGTTTGATGAGATGTAAATGTTACAAGGTTCGCAAAGCATTTTCAACAAACATTAAATCATATGTTTAACAATTTTCAGATATTTTATGCGGTTTGTTATCCGATATAGATTGTATTTCTTTTCTCTTTTATGTGAACAAATTTTATTTAATTACTTGGTCTTATTATCACACTACGTTTAATGAACAATCCAATAAATTATAAACCGATGAGAAAAACCTTTTGTAAAATGGA

>S1.3

FSEDFIRNRIHIIIMQNISTLNESEFYQNKSFIFSDIPKNIKWPFLFLLVIPLITIFGNILVGVSVYLERRLQNRFNYFLVSLAVSDFLCAILVMPVSTVKMVNAELTNGWPHQLCLAWYSLDVFFTATTIIHLCTISIDRYVALNNPLRFHQTKRKYSLFIKISISWLIPFGIACPLFLSSLKIETLNQEQSSFKGCGPNNAIFIMTAVIVTFILPLLIMMISYILTVKTIKNQTKNLDVFSHNGYSKSRLSQSEIYKKSVASTSLNTSIVNVHSKICEEKLSLLSISTDFSRRSLMSCSNLPTTPNRIQENNDCEKETNFDLKSKFCRLSRQMSIPFRKNTLRLHRNSSVNSERLRLCANVLLNSRLSVNSRRIATSDLSLRYIQKSRKAVQVLGIVFGLFVCCYLPFFVIYLCDFFCVSCQNITGWMIGYSEWIGYSASMMNPVVYHIFNPTFRRTFNRLVKCRCYKVGNFQRSGSFLS

>S1.3

TTTTCAGAAGACTTTATTAGAAATAGAATTCATATAATTATTATGCAGAATATATCCACCCTCAATGAATCAGAGTTCTATCAAAATAAATCATTTATTTTTTCAGATATTCCAAAAAATATTAAATGGCCATTTTTGTTCTTATTAGTGATACCTTTAATTACTATATTTGGAAATATTCTCGTTGGAGTGTCAGTTTATCTTGAAAGACGACTGCAGAATAGGTTCAACTATTTTCTTGTTTCTTTAGCTGTAAGTGACTTTTTATGTGCAATTTTGGTTATGCCGGTCTCCACAGTCAAAATGGTTAATGCTGAACTTACAAATGGATGGCCCCACCAACTTTGTTTGGCGTGGTATAGTTTGGATGTTTTTTTCACTGCTACAACAATAATTCATTTATGTACAATATCGATTGATCGATATGTTGCACTCAATAATCCATTACGCTTCCATCAAACCAAACGTAAATATTCTTTGTTCATCAAAATTTCAATTTCCTGGTTGATACCCTTTGGAATAGCTTGCCCCTTGTTTTTGTCATCGTTAAAAATCGAAACTCTAAATCAAGAACAAAGCTCATTCAAAGGATGTGGTCCAAATAATGCAATTTTTATTATGACAGCTGTGATTGTTACATTTATCTTACCATTATTAATTATGATGATATCGTACATTTTAACTGTCAAAACTATTAAAAACCAAACAAAAAACCTTGACGTATTTTCCCATAATGGCTATTCTAAAAGTCGACTAAGTCAATCAGAAATATATAAAAAATCTGTGGCTAGCACTAGCCTAAATACTTCCATTGTAAATGTTCATTCTAAGATTTGCGAAGAAAAATTAAGCTTATTATCAATATCAACTGATTTTTCTAGGCGATCCTTAATGAGTTGTAGCAACTTACCAACAACACCAAATAGAATACAAGAGAATAATGACTGTGAAAAAGAAACAAATTTTGATTTGAAAAGCAAATTCTGTCGACTGTCAAGACAAATGAGTATTCCATTTCGTAAAAATACTCTACGGCTTCACAGAAATAGTTCTGTCAATTCTGAACGATTGAGATTATGTGCAAATGTTCTTTTAAATAGCCGGCTATCAGTGAATTCTCGACGAATTGCTACTTCTGATCTTAGTCTAAGATATATTCAAAAAAGTCGAAAAGCGGTTCAAGTTTTGGGAATTGTTTTCGGCTTATTTGTTTGTTGTTATTTGCCGTTTTTTGTTATTTATCTCTGTGATTTTTTCTGTGTTTCATGTCAGAATATTACAGGTTGGATGATTGGTTATAGTGAATGGATTGGTTATTCCGCTTCAATGATGAATCCGGTGGTTTATCATATTTTCAACCCAACATTTCGACGAACATTCAATAGACTAGTAAAATGCCGATGTTATAAAGTCGGAAATTTTCAAAGATCTGGAAGTTTTCTTTCTTAGATTTTGTCAAA
